# VER-246608, a novel pan-isoform ATP competitive inhibitor of pyruvate dehydrogenase kinase, disrupts Warburg metabolism and induces context-dependent cytostasis in cancer cells

**DOI:** 10.18632/oncotarget.2656

**Published:** 2014-11-02

**Authors:** Jonathan D. Moore, Anna Staniszewska, Terence Shaw, Jalanie D'Alessandro, Ben Davis, Alan Surgenor, Lisa Baker, Natalia Matassova, James Murray, Alba Macias, Paul Brough, Mike Wood, Patrick C. Mahon

**Affiliations:** ^1^ Vernalis (R&D) Ltd, Granta Park, Cambridge, UK; ^2^ Current address: Horizon discovery, Cambridge Research Park, Waterbeach, Cambridge, UK

**Keywords:** Pyruvate dehydrogenase kinase, glycolysis, Warburg metabolism, Nov3r

## Abstract

Pyruvate dehydrogenase kinase (PDK) is a pivotal enzyme in cellular energy metabolism that has previously been implicated in cancer through both RNAi based studies and clinical correlations with poor prognosis in several cancer types.

Here, we report the discovery of a novel and selective ATP competitive pan-isoform inhibitor of PDK, VER-246608. Consistent with a PDK mediated MOA, VER-246608 increased pyruvate dehydrogenase complex (PDC) activity, oxygen consumption and attenuated glycolytic activity. However, these effects were only observed under D-glucose-depleted conditions and required almost complete ablation of PDC E1α subunit phosphorylation. VER-246608 was weakly anti-proliferative to cancer cells in standard culture media; however, depletion of either serum or combined D-glucose/L-glutamine resulted in enhanced cellular potency. Furthermore, this condition-selective cytostatic effect correlated with reduced intracellular pyruvate levels and an attenuated compensatory response involving deamination of L-alanine. In addition, VER-246608 was found to potentiate the activity of doxorubicin. In contrast, the lipoamide site inhibitor, Nov3r, demonstrated sub-maximal inhibition of PDK activity and no evidence of cellular activity.

These studies suggest that PDK inhibition may be effective under the nutrient-depleted conditions found in the tumour microenvironment and that combination treatments should be explored to reveal the full potential of this therapeutic strategy.

## INTRODUCTION

It is becoming increasingly recognized that the aberrant metabolic programme found in cancer cells is not simply a secondary consequence of the altered expression/activity of the protein products of cancer-associated genes, but rather an essential component in the process of cellular transformation. Studies by Otto Warburg in the 1920s provided the foundation for this field of cancer biology with his observation of increased glycolytic rate coupled with increased glucose uptake in ascites tumour cells, even in the presence of sufficient oxygen to oxidise glucose through aerobic respiration [1]. Indeed, this ‘aerobic glycolysis’ phenotype of cancer cells has been exploited in the development of 2-[18F]fluorodeoxyglucose positron emission tomography (FDG-PET) for the visualisation and monitoring of a range of different tumour types, demonstrating the existence of this phenomenon in the clinical setting [2]. Over the past decade, a growing body of literature has provided evidence for a direct link between common oncogenic genetic alterations and the establishment of an altered metabolic profile [3-6].

These findings have led to a surge of interest in the identification of factors that represent critical nodes in metabolic pathways that are central to the maintenance of the altered metabolic phenotype of cancer cells. Several recent studies have provided evidence which points to pyruvate dehydrogenase kinase (PDK) as one such factor. PDK, a member of the GHKL ATPase/kinase superfamily, regulates the activity of the pyruvate dehydrogenase complex (PDC). The PDC represents a central control point in cellular energy metabolism in that it links glycolysis with the TCA cycle by catalysing the oxidative decarboxylation of pyruvate to acetyl-CoA in the matrix of the mitochondria. Therefore, the PDC controls the degree to which pyruvate is utilised for ATP generation via oxidative phosphorylation as opposed to alternative fates such as oxidation to L-lactate or transamination to L-alanine. The four mammalian isoforms of PDK (PDK-1,2,3 & 4) reduce PDC activity through phosphorylation of specific serine residues in the E1α subunit (pyruvate dehydrogenase). Conversely, reactivation of the PDC is controlled by the activity of two E1 phosphatase isozymes, PDP-1 and PDP-2 [7].

PDK isozymes have been shown to be over-expressed in clinical cancer specimens and this augmented expression has been correlated with poor prognosis as well as drug resistance [8-10]. In addition, PDK gene expression has been shown to be up-regulated under conditions relevant to the tumour microenvironment such as hypoxia (PDK-1 & PDK-3) or by inactivating mutations in common tumour suppressor genes such as p53 (PDK-2) and pRB (PDK-4) [11,12]. Previous studies employing RNAi have provided evidence for a survival role for PDK-1 under hypoxic conditions as well as its importance in maintaining the glycolytic phenotype of cancer cells [13-15]. DCA, a weak inhibitor of PDK, has previously been used to study the role of PDK in cancer; however, interpretation of these studies has been complicated by conflicting data and the lack of specificity of this agent for PDK [16-20].

Here, we report the discovery of a novel pan-isoform ATP competitive inhibitor of PDK, VER-246608. Using this compound we provide evidence to support the strategy of targeting the ATP site of PDK in favour of the lipoamide domain. In addition, we demonstrate that inhibition of PDK with VER-246608 can disrupt the glycolytic phenotype of cancer cells, but only under nutrient-depleted conditions and that almost complete ablation of biomarker levels were required to observe these effects. Furthermore, we identify culture conditions and combinatorial treatments which enhance the therapeutic efficacy of this agent as well as defining the metabolic basis for these observations.

## RESULTS

### VER-246608 is a potent and selective pan-isoform small molecule inhibitor of pyruvate dehydrogenase kinase

**Figure 1:**
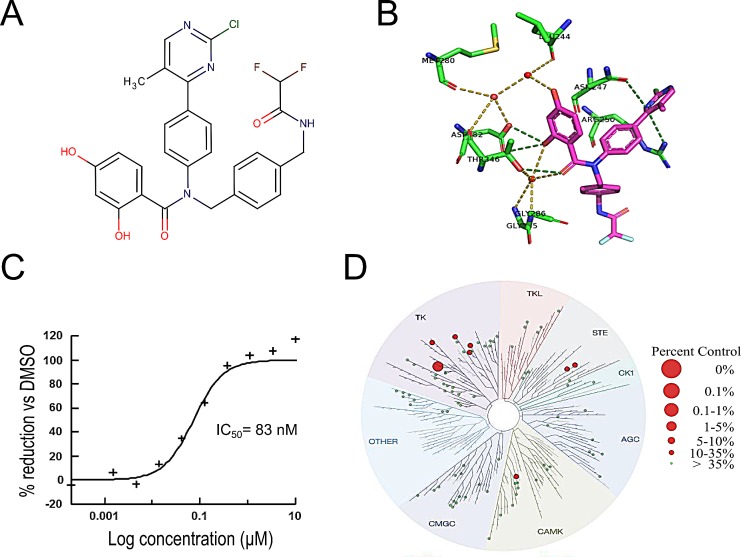
VER-246608 is a potent and selective ATP-competitive inhibitor of PDK A, molecular structure of VER-246608 (MW, 552.96 kDa). B, X-ray crystal structure (2.6 Ǻ) of VER-246608 in the ATP binding site of PDK-2. Water molecules are represented as red spheres and dashed lines indicate hydrogen bonds. C, representative fluorescence polarization assay binding curve demonstrating the ability of VER-246608 to compete with a fluorescein-labelled probe for the ATP binding site of PDK-1. D, treespot interaction map illustrating the selectivity profile of VER-246608 against a panel of 96 kinases spanning all seven kinase family groups from a *scan*EDGE screen (Discoverx).

**Figure 2:**
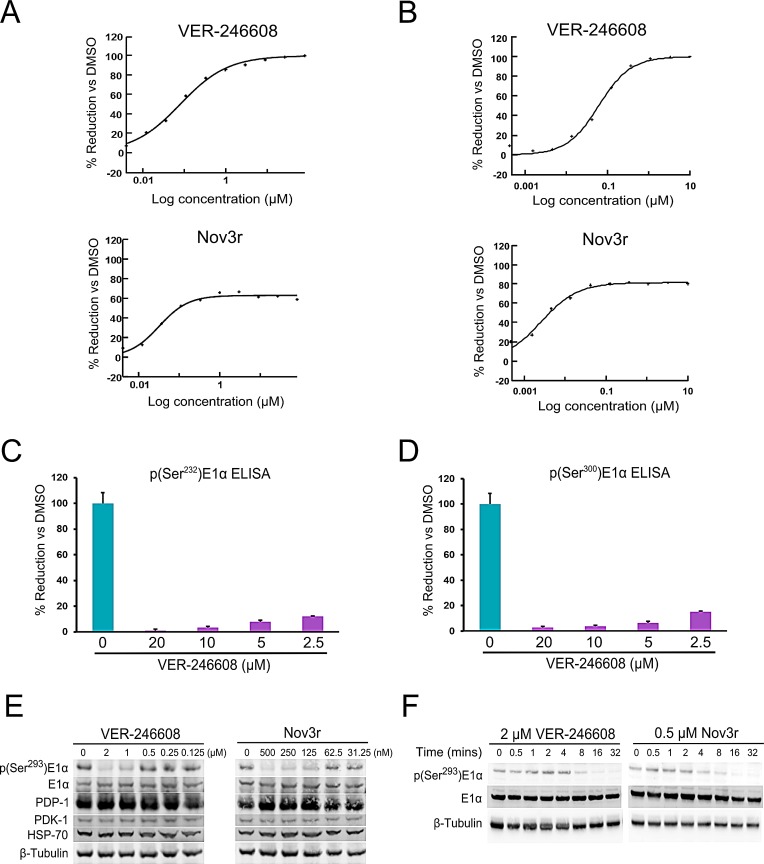
Comparison of the biochemical and cellular potency of VER-246608 and Nov3r A, representative MSD ELISA IC_50_curves illustrating the concentration dependent effect of VER-246608 and Nov3r on p(Ser^293^)E1α levels in PC-3 cells. B, representative DELFIA-based functional assay IC_50_curves illustrating the concentration dependent effect of VER-246608 and Nov3r on PDK-1 enzyme activity. C and D, PC-3 cells were seeded into 6 well plates and treated with the indicated concentration of VER-246608 for 90 mins, followed by cell lysis and the determination of p(Ser^232^)E1α (C) and p(Ser^300^)E1α (D) levels using commercially available ELISA assay kits (Abcam). Results are expressed as a percentage of DMSO control values. Error bars represent standard deviation of the mean. E and F, PC-3 cells were seeded into 60 mm culture dishes and treated with the indicated concentrations of VER-246608 and Nov3r along with DMSO control for 90 minutes (E) or for varying time periods (F). Cell lysates were then subjected to immunoblot analysis with antibodies against the indicated proteins. β-tubulin served as a loading control.

VER-246608 was discovered through a combination of NMR-based fragment screening and structure-guided medicinal chemistry to enhance compound potency and selectivity (Fig. 1A). The X-ray crystal structure of VER-246608 bound to PDK-2 (2.6 Ǻ) reveals that the resorcinol moiety forms a series of direct and water-mediated hydrogen bonds to various residues within the PDK-2 ATP binding site, including Asp282 and Thr346. The chloro-methylpyrimidine also forms additional contacts to the side chains of residues Asn247 and Arg250 with the di-fluoro pointing towards the open solvent region (Fig. 1B). Fig. 1C shows a representative fluorescence polarization assay binding curve demonstrating the ability of VER-246608 to compete with a fluorescein-labeled probe for the ATP binding site of PDK-1. VER-246608 demonstrated similar potency across all four PDK isoforms in a DELFIA-based enzyme functional assay in the sub 100 nM range (Table 1). Consistent with previous reports, the lipoamide site-binding compound Nov3r was found to stimulate the activity of PDK-3 and PDK-4 (but not PDK-1 or PDK-2) by approximately 2-fold in this assay (data not shown)[21]; however, in a modified assay incorporating E1/E2 as the substrate Nov3r generated an IC_50_value of 3 nM. In general, there was good agreement between the functional assay and binding affinity data for VER-246608. The marked selectivity of VER-246608 for PDK-1 versus HSP-90 (a fellow member of the GHKL kinase family sharing substantial homology with PDK in the ATP binding pocket) is demonstrated both in terms of the relative FP IC_50_values (>1000 fold selectivity) and the lack of a detectable induction of HSP-70 (a well-established biomarker for HSP-90 inhibition) in PC-3 cells (Table 1 and Fig. 2C). In order to assess the selectivity of VER-246608, a ScanEDGE screen (DiscoverX, USA) was performed at a concentration of 10 μM against a panel of 97 kinases spanning all 7 kinase family groups. From the results of this screen, ligand binding to the respective kinases was reduced to ≤ 10% of control values (100% of control values indicating no evidence of compound binding) for only one of the kinases in the panel and between 10 and 35% for a further seven. Therefore, VER-246608 demonstrated significant or partial binding to only 8% of the kinases on the panel at a concentration that achieves complete inhibition of PDK activity in biochemical assays, suggesting a reasonable selectivity profile for this compound (Fig. 1D).

**Table 1:** Summary of the biochemical and cellular potency data for VER-246608 and Nov3r. ITC = Isothermal calorimetry; FP = Fluorescence polarization; IC_50_ = the compound concentration which achieves a 50% reduction in signal compared to DMSO control. EC_50_ = the compound concentration which achieves a 50% increase in signal compared to DMSO control. The mean value of the indicated number of independent determinations are given. ND = not determined

Assay	VER-246608 (nM)	Nov3r (nM)
***In vitro* functional (E1), IC_50_**		
PDK-1	35 (n=5)	ND
PDK-2	84 (n=2)	ND
PDK-3	40 (n=3)	ND
PDK-4	91 (n=4)	ND
***In vitro* functional (E1/E2), IC_50_**		
PDK-1	ND	3 (n=2)
***In vitro* affinity**		
PDK-1 ITC, Kd	150 (n=2)	8 (n=2)
HSP-90 FP, IC_50_	>100 μM (n=4)	>200 μM (n=2)
PDK-1 FP, IC_50_	79 (n=3)	83 (n=10)
**PD marker (ELISA)**		
p(Ser^293^)E1α (0% FCS), IC_50_	266 (n=4)	5 (n=3)
p(Ser^293^)E1α (10% FCS), IC_50_	1.45 μM (n=1)	ND
HSP-70, EC_50_	>80 μM (n=2)	>80 μM (n=2)

In terms of cellular biomarker modulation, both VER-246608 and Nov3r suppressed the phosphorylation of the Ser^293^residue of E1α (phosphorylated by all four PDK isozymes) with IC_50_values of 266 and 55 nM, respectively, suggesting efficient target engagement for both compounds. Inspection of the curves from the p(Ser^293^)E1α MSD ELISA analysis reveals that although VER-246608 can achieve complete suppression of p(Ser^293^)E1α levels in cells, the degree of suppression achieved by Nov3r reaches a plateau at an approximately 60% reduction compared to DMSO control values (Fig. 2A). This sub-maximal suppression of p(Ser^293^)E1α levels was also observed in other cell lines (for example, 80% in ES-2 cells). This observation would appear to be at least partly explained by the inability of Nov3r to achieve complete inhibition of PDK activity *in vitro* (Fig. 2B). VER-246608 also demonstrated a similar degree of potency with regard to its ability to suppress the phosphorylation of the two remaining serine residues targeted by the PDK isozymes, S232 (Fig. 2C) and S300 (Fig. 2D). The ability of VER-246608 to reduce cellular p(Ser^293^)E1α levels did not appear to be due to alterations in the expression level of other proteins which could influence the phosphorylation state of the PDC such as PDK-1, E1α and PDP-1 (Fig. 2E). Interestingly, both VER-246608 and Nov3r required a similar period of time (16 min) to achieve maximal biomarker suppression (Fig. 2F). Another compound, VER-246520, a close analogue of VER-246608, demonstrated a comparable biochemical and cellular potency profile as well as a similar binding mode within the ATP site of PDK-2 (Supplementary Fig. S1).

### Inhibition of PDK activity with VER-246608 results in a reversal of Warburg metabolism

To confirm that PC-3 cells demonstrate a Warburgian (or glycolytic) phenotype, we investigated the ability of this cell line to proliferate in media containing either D-glucose or D-galactose (requires mitochondrial respiration to be metabolised) as a fuel source. As can be seen from Supplementary Fig. S2A, PC-3 cells demonstrated a substantial reduction in growth in D-galactose versus D-glucose containing media. K562 and Jurkat leukemia cell lines were also identified as highly glycolytic based on this analysis which contrasts with the oxidative cell line MDA-MD-453 [22]. Treatment of these cell lines with 20 μM VER-246608 had little to no effect in either media, indicating that inhibition of PDK does not rescue growth in D-galactose containing media.

**Figure 3:**
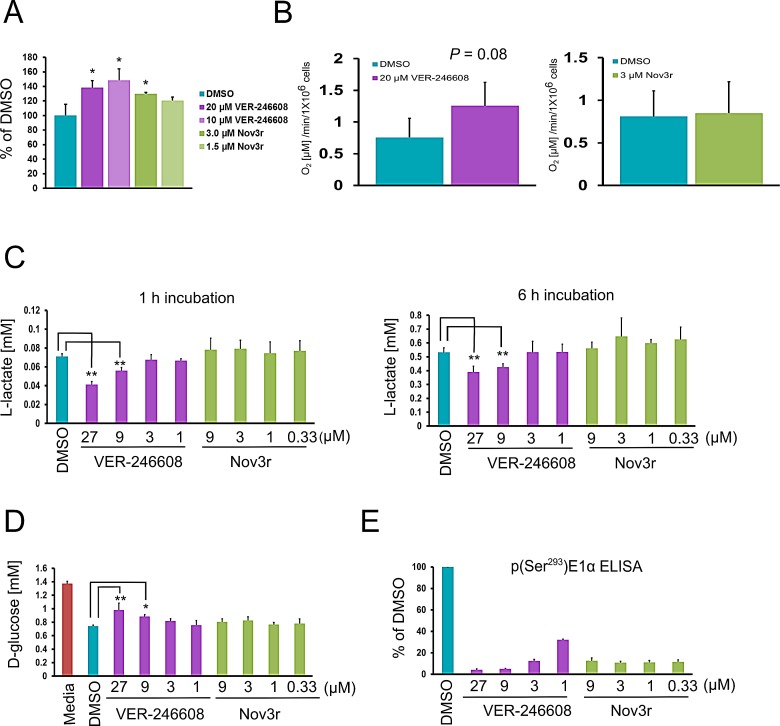
VER-246608 disrupts Warburg metabolism A, PC-3 cells were treated with the indicated concentrations of VER-246608 and Nov3r for 2 h followed by analysis of pyruvate dehydrogenase (PDH) activity in cell lysates using a commercially available ELISA-based assay kit (Abcam). Results are expressed as a percentage of the PDH reaction rate in DMSO treated cells. B, PC-3 cells in suspension were pre-treated with either DMSO, 20 μM VER-246608 or 3 μM Nov3r for 1 h followed by kinetic measurement of oxygen levels using a Hansatech ‘oxytherm’ oxygen electrode system. C, D and E, PC-3 cells were seeded into 96 well plates at a density of 1×10^4^cells per well in 96 well plates. The following day the cells were treated with the indicated concentrations of VER-246608 and Nov3r (in RPMI-1640 media containing 0.25 g/L D-glucose and 2% dialyzed FCS), followed by the removal of culture media for the analysis of L-lactate (C) and D-glucose (D) levels. The same cells used for the metabolite analysis were analyzed for p(Ser^293^)E1α levels by MSD ELISA (E). Results are representative of 2-3 independent experiments performed in triplicate (A and B) or quadruplicate (C-E). Error bars represent standard deviation of the mean (*, *P* < 0.05; **, *P* < 0.01).

As expected, the observed suppression of p(Ser^293^)E1α levels in cells treated with VER-246608 and Nov3r resulted in an increase in PDC activity in both PC-3 and K562 cells, with the magnitude of the increase being greater for VER-246608 (Fig. 3A and Supplementary Fig. S2C). In order to determine whether this increase in PDC activity resulted in a change in mitochondrial respiration, we measured the effect of VER-246608 and Nov3r on oxygen consumption rates in PC-3 cells. Treatment of PC-3 cells with 20 μM VER-246608 resulted in a 66% increase in the rate of oxygen consumption, whereas Nov3r had no discernible effect at concentrations which exceeded those required to achieve maximal biomarker suppression (≥ 1μM) (Fig. 3B and Fig. 2B).

We next investigated the effect of these compounds on glyoclytic rate by measuring L-lactate production and D-glucose consumption. Initial experiments revealed that it was necessary to deplete D-glucose levels in the media to below 0.5 g/L before any change in media L-lactate levels could be observed in compound treated cells. Treatment of PC-3 cells with 9 μM and 27 μM VER-246608 resulted in a 21% and 42% reduction, respectively, in media L-lactate levels following a 1 h incubation; however, no change was observed with Nov3r at all concentrations tested (Fig. 3C). A similar result was obtained following a 6 h incubation; however, the magnitude of the reduction was slightly reduced, indicating the induction of a compensatory cellular response.

VER-246608 also decreased D-glucose consumption at the same concentrations that resulted in reduced L-lactate production (Fig. 3D). Analysis of biomarker levels revealed that that a > 88% reduction in E1α Ser^293^phosphorylation was required to observe these effects (Fig. 3E). The fact that Nov3r did not achieve this level of biomarker suppression suggests that this is the underlying reason for its lack of activity. In agreement with the above observations, ^13^C-NMR analysis demonstrated that treatment of Jurkat cells with 20 μM VER-246608 resulted in a 25% and 23% decrease in the rate of L-lactate production and D-glucose consumption, respectively (Supplementary Fig. S2D). The ability of VER-246608 to modulate glycolytic activity in the non-transformed hTert immortalized human foreskin fibroblast (HFF) cell line was also studied. VER-246608 treatment resulted in a decrease in extracellular L-lactate and D-glucose levels in this cell line; however, the magnitude of this reduction was less pronounced compared to PC-3 cells and only occurred at the top concentration (27 μM) tested (Supplementary Fig. S2B). As with PC-3 cells, Nov3r did not alter glycolytic activity in HFF cells at any of the concentrations tested.

### VER-246608 induces context-dependent cytostasis in cancer cells

Having established that VER-246608 can achieve biological effects in cancer cells that are consistent with a reversal of Warburg metabolism, we next sought to investigate whether this compound is cytotoxic to cancer cells. We found that VER-246608 was weakly cytotoxic (based on reduced cell mass using the Sulforhodamine B assay) to a set of cell lines selected for on the basis of having a high level of PDK-1 mRNA expression (PC-3 & SW-1088) based on publically available (DTP, NCI-60) microarray data, as well as additional cell lines (UM-22B & MEF) which have been reported to be sensitive to PDK-1 knock-down under hypoxic conditions [13, 14]. We hypothesized that the supraphysiological D-glucose concentrations present in standard cell culture media may be limiting the effect of PDK inhibition due to elevated intracellular pyruvate concentrations. Therefore, we decided to explore conditions of limited nutrient availability or conditions which influence nutrient uptake such as serum-derived growth factor levels. We found that in all four of these cell lines, VER-246608 demonstrated increased efficacy under reduced serum conditions. Compound potency was also increased in the three cancer cell lines (but not in the non-transformed MEF cell line) when the cells were incubated in D-glucose-depleted media (Supplementary Table S1). In contrast to previous RNAi based studies, no evidence of augmented compound potency was observed under hypoxic (0.1% O_2_) conditions. Incubation of PC-3 cells under milder hypoxic conditions (1% O_2_) also showed no effect on compound potency compared to standard culture conditions (data not shown). A similar profile of enhanced compound potency under glucose or serum-depleted conditions was observed with VER-246520 (Supplementary Table S2).

**Figure 4:**
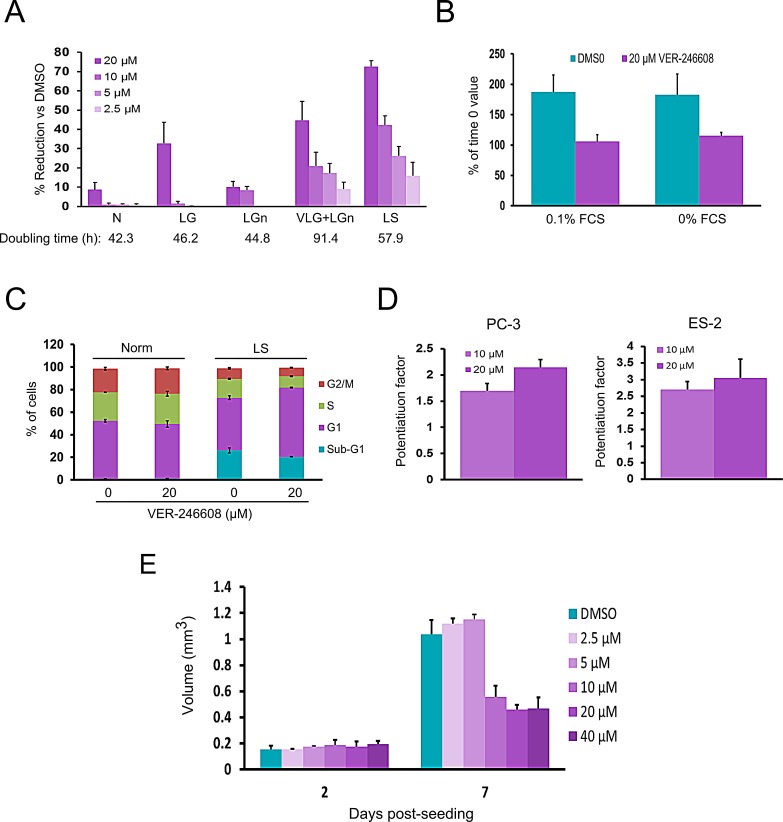
VER-246608 induces context-dependent cytostasis in PC-3 cells A, Cells were seeded into 96 well cell culture plates (2×10^3^ per well). The following day, the cells were treated with the indicated concentrations of VER-246608 for 120 h followed by cell mass determination using the Sulforhodamine B assay. Results are expressed as a percentage decrease versus DMSO control values. Norm = D-glucose and L-glutamine free media supplemented with 10% FCS, 2 g/L D-glucose and 0.3 g/L L-glutamine; VLG (very low glucose) = 0.1 g/L D-glucose; LGn (low L-glutamine) = 15 mg/L L-glutamine; LS (low serum) = 0.5% FCS. B, Cells were treated with either DMSO or 20 μM VER-246608 in media containing either 0% or 0.1% FCS for 120 h followed by cell mass determination using the Sulforhodamine B assay. Results are expressed as the percentage change versus time 0 (just prior to compound addition) values. C, cell cycle and sub-G1 analysis of cells treated with either DMSO or 20 μM VER-246608 for 48 h prior to PI staining and FACS analysis. D, Analysis of the potentiation of doxorubicin by VER-246608 in PC-3 and ES-2 cells. E, Effect of the indicated concentrations of VER-246608 on the growth of PC-3 spheroids. Experiments were carried out in triplicate. Error bars represent standard deviation of the mean.

As cancer cell lines are known to utilize L-glutamine as a means to fuel anaplerotic reactions [23], the possibility that combined depletion of D-glucose and L-glutamine might further influence cytotoxicity was explored. Reduction of L-glutamine levels to 15 mg/L (in combination with D-glucose depletion) resulted in both reduced doubling time and enhanced potency of VER-246608 compared to depletion of either of these fuel sources alone (Fig. 4A). As the cells only completed one doubling during the experiment under this condition, the 50% reduction in cell mass achieved with 20 μM VER-246608 vs DMSO control values equates to an almost complete inhibition of cell growth. VER-246608 also achieved a substantial reduction in growth (70% at a concentration of 20 μM) under serum depleted conditions (Fig. 4A), with a complete suppression of growth observed in either serum-free media or media containing very low levels (0.1%) of serum (Fig. 4B). In contrast, Nov3r demonstrated no evidence of cytotoxicity/cytostasis-induction under any of the above culture conditions up to a top concentration of 80 μM.

In order to attempt to identify cancer associated genetic alterations which predict sensitivity to PDK inhibition we decided to study cell lines harboring oncogenic mutations in genes which are linked with a highly glycolytic phenotype (KRAS) or have been previously reported to demonstrate sensitivity to PDK-1 knock-down (BRAF) [24, 25]. No evidence of enhanced sensitivity to VER-246608 was observed for cell lines possessing these genotypes although, surprisingly, the oxidative wild-type KRAS breast cancer cell line MDA-MB-453 displayed the greatest degree of sensitivity, with reduced cell mass observed even under normal media culture conditions (Supplementary Fig. S3A and S3B). In contrast, VER-246608 was largely inactive against HFF cells under all of the conditions tested (Supplementary Fig. S3B).

The nature of the above condition-selective cytotoxicity was investigated by PI/FACS analysis of PC-3 cells that had been treated with either DMSO or 20 μM VER-246608 under either standard of serum-depleted conditions. This analysis revealed an increased fraction of cells in the G1 phase of the cell cycle in compound treated cells under serum-starved conditions (Fig. 4C). No evidence of apoptosis induction was observed based on a comparison of the percentage of cells in the sub-G1 fraction under both conditions.

As defective oxidative phosphorylation capacity has been previously linked with sensitivity to treatment with DCA [17], the ability of VER-246608 to potentiate the cytotoxicity of cancer therapeutic agents (Doxorubicin, BEZ235, Cisplatin and 3-Bromopyruvate) which are known to inhibit mitochondrial function was evaluated. This analysis revealed that VER-246608 achieved a 2-fold and 3-fold potentiation of doxorubicin in PC-3 and ES-2 cells, respectfully (Fig. 4D). No evidence of potentiation of the remaining three agents by VER-246608 was observed.

We next investigated the effect of PDK inhibition on cancer cell growth in a 3D culture format. As can be seen from Fig. 4E, an approximately 50% reduction in spheroid volume was achieved at concentrations of 10 μM and above, suggesting an increase in compound potency compared to monolayer growth. Analysis of the media demonstrated no evidence of the presence of either LDH or soluble caspase-cleaved Cytokeratin 18 fragments, indicating that the reduction in spheroid size in the presence of VER-246608 did not result from the induction of cell death or apoptosis.

### VER-246608 modulates key metabolite levels under austere culture conditions

**Figure 5:**
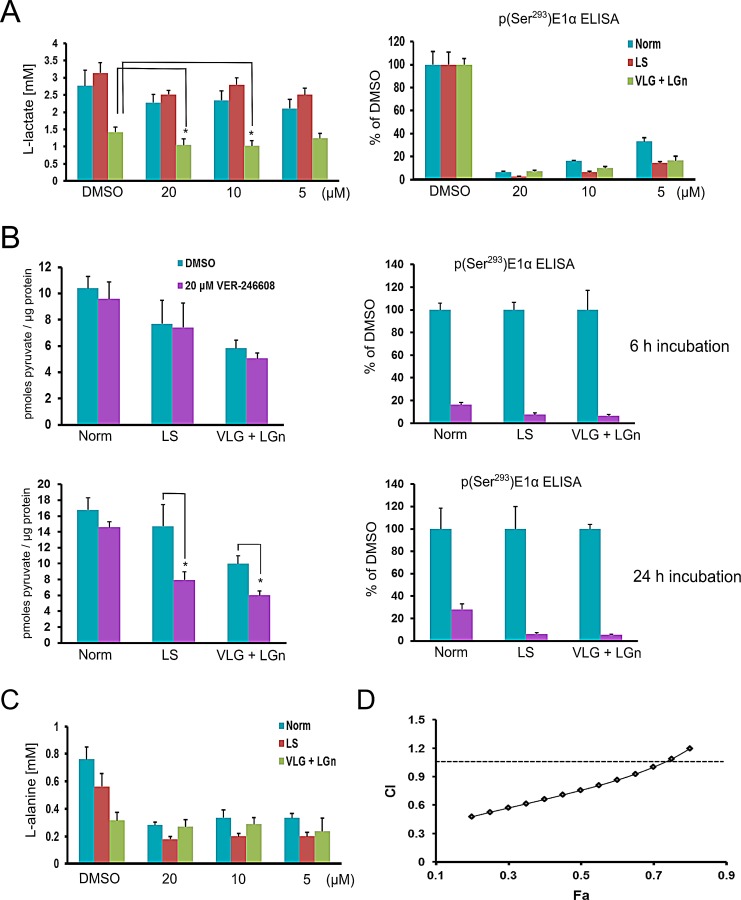
VER-246608 modulates key metabolite levels in PC-3 cells under nutrient and serum-depleted conditions A, Analysis of L-lactate levels in the media of PC-3 cells treated with 0, 5, 10 and 20 μM VER-246608 followed by an incubation period of 24 h (under the indicated culture conditions). At the end of the experiment, the remaining media was aspirated and the cells frozen at −80°C for subsequent determination of p(Ser^293^)E1α levels by MSD ELISA. B, Cells were treated with either DMSO or 20 μM VER-246608 under the indicated culture conditions for 6 and 24 h prior to lysate preparation and pyruvate quantification. The same lysates were used to determine protein concentrations and pSer^293^E1α levels by MSD ELISA. C, The same media from (A) was used to determine L-alanine levels. D, Combination index (CI) analysis of PC-3 cells treated with either VER-246608, β-chloro-alanine or a fixed combination ratio of both compounds. Results are representative of 2-3 independent experiments performed in quadruplicate (A-C) or triplicate (D). Error bars represent standard deviation of the mean (*, *P* < 0.01).

In order to better understand the context-dependent cellular activity of VER-246608 we studied the modulation of key metabolites which would be expected to be influenced by changes in PDK activity. The production of one such metabolite, L-lactate, is an indicator of glycolytic activity, a process which is known to be central to supporting rapid proliferation [26]. Thus, the effect of VER-246608 on extracellular L-lactate levels was investigated under the same conditions employed in the cytotoxicity studies. We found that L-lactate production was not altered in VER-246608 compared to DMSO treated cells cultured in either normal or low serum media with basal L-lactate production being similar under both of these conditions (Fig. 5A). However, there was a substantial reduction in extracellular L-lactate levels when cells were cultured in D-glucose/L-glutamine-depleted media with a further reduction observed in the presence of 10 and 20 μM VER-246608, correlating with a >70% reduction in p(Ser^293^)E1α levels.

Pyruvate is a central metabolite both in bridging glycolysis with aerobic respiration as well as a substrate for the PDC reaction. Therefore, we next investigated the effect of VER-246608 on intracellular levels of this metabolite under the relevant culture conditions. Although basal pyruvate levels were reduced after a 6 h incubation period in both the low serum and D-glucose/L-glutamine depleted conditions, treatment with 20 μM VER-246608 did not alter pyruvate levels further (Fig. 5B). However, extending this incubation period to 24 h revealed a marked reduction in intracellular pyruvate levels in the VER-246608 treated cells under both D-glucose/L-glutamine and serum depleted conditions whereas no change was observed under standard culture conditions. Notably, based on the analysis of p(Ser^293^)E1α levels there was an apparent increase in compound potency under the austere conditions compared to standard media.

We hypothesized that alternative sources of pyruvate in the cell are being utilized in an attempt to restore homeostasis of this metabolite and that this alteration in metabolic flux may become limiting under nutrient depleted conditions. We used ^1^H-NMR metabolomic analysis in an attempt to identify possible candidate mechanisms for this compensatory response. This analysis revealed reductions in the intracellular levels of L-alanine and the branched chain amino acids leucine and valine (which can serve as metabolic precursors of L-alanine) in cells treated with PDK inhibitors (data not shown). To confirm these observations we measured extracellular L-alanine levels in cells treated with either DMSO or increasing concentrations of VER-246608. Incubation of cells in either serum or D-glucose/L-glutamine-depleted media resulted in a reduction in baseline extracellular L-alanine production which was particularly marked in the D-glucose/L-glutamine depleted media (Fig. 5C). Interestingly, and in accord with the ^1^H-NMR data, treatment with 20 μM VER-246608 resulted in a further substantial decrease in extracellular L-alanine levels under both standard and low serum culture conditions, compared to DMSO treated cells. One interpretation of these observations is that L-alanine is being utilized to compensate for loss of cytoplasmic pyruvate resulting from increased PDC activity through alanine aminotransferase (ALAT) catalyzed transamination. This hypothesis was supported by the observation of synergy between the ALAT inhibitor β-Chloro-alanine and VER-246608 under normal culture conditions based on combination index (CI) analysis (Fig. 5D). Furthermore, we found that 20 μM VER-246608 could potentiate β-Chloro-alanine in PC-3 cells (potentiation factor = 1.8 ± 0.06). Therefore, we propose that the reduction in pyruvate levels in VER-246608 treated cells under the above austere conditions is due to a combination of increased compound potency (and therefore an elevated rate of pyruvate oxidation by the PDC) coupled with decreased availability of L-alanine to compensate for this reduction. Thus, the cell can no longer maintain pyruvate homeostasis leading to attenuated glycolytic rate and cell cycle arrest (graphically illustrated in supplementary Fig. S4).

## DISCUSSION

The growth of interest in cancer metabolism over the past decade has led to the identification of several novel therapeutic targets that play important roles in establishing and maintaining the aberrant metabolic phenotype of cancer cells [27]. Several key findings over this period have led to growing support for the proposal that this phenomenon acts as a driver of both carcinogenesis and tumour progression, rather than a dispensable secondary consequence of common cancer-associated genetic alterations.

PDK has emerged as factor which fits well into the above paradigm in that it has been implicated in both the process of cellular transformation as well as a factor that is deregulated following the activation of common oncogenic signaling pathways [28, 29]. These findings, in addition to previous reports which demonstrate the therapeutic effect of depleting individual PDK isoforms, paint an encouraging picture for PDK as a target for cancer therapy.

Initial efforts at developing PDK inhibitors focused on targeting the lipoamide binding domain. These compounds, notably AZD7545 and Nov3r, were developed for the treatment of type 2 diabetes, with AZD7545 progressing to a phase 1 clinical trial [30, 31]. One issue with these compounds is that they demonstrate differential activity towards different PDK isoforms and actually stimulate PDK-4 activity in biochemical assays [32]. Our studies have also revealed that Nov3r, in contrast to VER-246608, cannot achieve complete suppression of PDK enzyme activity or p(Ser^293^)E1α biomarker levels in cells. To date, DCA, which binds to the allosteric pyruvate binding site on PDK, is the only small molecule that has been used to study the role of PDK in cancer; however, this agent is a very weak inhibitor requiring concentrations in the 5-20 mM range to observe modulation of glycolytic activity and cytotoxicity. As with the lipoamide site inhibitors, DCA demonstrates differing activity towards the four mammalian PDK isoforms and also a lack of specificity for PDK [33, 13]. In our hands, knock-down of PDK-1 or PDK-2 did not achieve measurable alterations in E1α phosphorylation and could therefore not be used as an approach to complement our small molecule inhibitor studies.

Here, we describe the discovery of a novel ATP-site binding pan-isoform PDK inhibitor, VER-246608. Initial experiments revealed that this compound demonstrated several biological effects which are consistent with a PDK mediated mechanism of action. In contrast to previous RNAi based studies, inhibition of glycolytic activity was only observed in D-glucose depleted media. Even under these conditions, almost complete suppression of p(Ser^293^)E1α levels were required to observe this effect. A likely explanation for this observation is the considerable capacity of cells to maintain homeostasis of pyruvate levels due to the crucial role played by this metabolite in central carbon metabolism. In addition, competition for pyruvate from lactate dehydrogenase due to its cytoplasmic localization and frequent over-expression in cancer cells, may limit the amount of pyruvate that is available to the PDC even when fully activated [34].

In accord with the above findings we observed that VER-246608 reduced cell mass with enhanced potency when cells were cultured in either nutrient or serum-depleted media. The nature of VER-246608 mediated cytotoxicity appeared to be predominantly cytostatic (cell cycle arrest at G1) with no evidence of apoptosis induction as assessed by sub-G1 analysis and a lack of an effect on caspase-3/7 activity (data not shown). Additionally, combination studies with mitochondriotoxic cancer therapeutic agents revealed that VER-246608 potentiated the cytotoxicity of doxorubicin. This finding contrasts with a report which found that DCA actually antagonized the activity of this agent [35].

Our efforts to link sensitivity to PDK inhibition with a particular oncogenic mutation status have been unsuccessful to date. These studies could clearly be further extended to examine additional oncogenic mutations linked with a glycolytic phenotype and sensitivity to glucose depletion such as mutant p53 and PTEN deficiency [36]; however, the possibility should be considered that the composition of the extracellular milieu may prove to be a better predictor of sensitivity rather than genotype *per se* (even if they are causally linked).

The metabolic basis for the context-dependent cytostatic effect of VER-246608 was also explored. Significantly, VER-246608 treatment was found to result in a substantial reduction in intracellular pyruvate levels under serum or nutrient-depleted conditions, whereas no effect was observed under standard culture conditions. The additional observation that L-alanine levels in the media were reduced under these austere conditions with a further reduction in the presence of VER-246608 under standard and low serum conditions provided the possibility that altered flux between pyruvate and L-alanine is being utilized as a compensatory response. Furthermore, the observed synergy between VER-246608 and the ALAT inhibitor β-Chloro-alanine suggests that this mechanism operates through blocking the ALAT catalyzed synthesis of pyruvate as opposed to the reverse reaction as, in this case, a synergistic effect is more readily explained by the fact that both compounds would work to reduce pyruvate levels through reduced synthesis and increased oxidation.

Although we are yet to obtain *in vivo* efficacy data for our compounds, it is tempting to speculate that certain characteristics of solid tumours may present the opportunity for additional therapeutic effects. For example, reducing lactate production through PDK inhibition may lead to a reduction in the acidification of the tumour microenvironment which has been linked with metastasis promotion and immune evasion [37]. In addition, the recent reports of metabolic symbiosis between tumour cells as well as between tumour cells and stromal components might also be disrupted through PDK inhibition [38, 39].

In summary, our findings have highlighted the challenging nature of PDK as a target for cancer therapy by demonstrating the requirement for compound potency levels that achieve almost complete suppression of PDC E1α phosphorylation in a concentration range that would allow realistic progression to animal studies. This conclusion was further strengthened by our observation that the lack of cytotoxicity observed with the lipoamide site inhibitor, Nov3r, appeared to correlate with its inability to achieve this critical degree of inhibition of PDK activity.

However, our observation that VER-246608 can effectively disrupt Warburg metabolism as well as substantially reduce intracellular pyruvate levels under austere culture conditions, provides evidence that targeting PDK may present an effective strategy to ‘normalise’ tumour metabolism. In addition, our demonstration that VER-246608 inhibits proliferation under conditions which more closely mimic the intratumoral environment both in monolayer and 3D culture formats provides hope that this therapeutic strategy may prove to be effective in an *in vivo* context as well as suggesting that a reasonable therapeutic window may be achievable. Furthermore, the ability of VER-246608 to potentiate the activity of doxorubicin suggests that combinatorial treatments may also prove to be a fruitful strategy to pursue with PDK inhibitors.

## Materials and Methods

### Cell lines, culture and reagents

All cell lines were obtained from the American Type Culture Collection (ATCC), grown in their recommended culture media supplemented with 10% FBS and antibiotics (penicillin / streptomycin) and passaged for < 4 months before reviving a low passage stock and screened for mycoplasma infection. The synthesis of VER-246608 and VER-246520 is described in the supplementary methods. β-Chloro-L-alanine, 2-deoxyglucose and 3-bromopyruvate (Sigma) were dissolved in water. Cisplatin (Selleckchem) was dissolved in saline solution (0.1% (w/v) NaCl). BEZ235 (Selleckchem) & doxorubicin (Calbiochem) were dissolved in DMSO. Media containing depleted levels of D-glucose and/or L-glutamine were prepared by their addition to media lacking these components (Life Technologies). Normal media contained D-glucose and L-glutamine at a final concentration of 11 mM (2 g/L) and 2 mM (0.3 g/L), respectively.

### Immunoblotting

Cell lysates were prepared by washing cells with PBS followed by the addition of lysis buffer (2% SDS, 50 mM TRIS-pH 6.8) directly to the well. Equal amounts of lysate were loaded onto pre-cast 4-12% SDS-PAGE gels (Life technologies) followed by transfer to PVDF membrane. The membranes were blocked for 1 h in TBST containing 5% non-fat dried milk, followed by a 2 h incubation with the relevant primary antibody: PDH phospho(Ser^293^)E1α (Genescript, custom rabbit polyclonal antibody), 1:600; PDH E1α (Abcam), 1:1000; PDP-1 (Sigma), 1:2000; PDK-1 (ENZO), 1:1000; HSP-70 (Santa Cruz), 1:5000; β-tubulin (Cell signaling technology), 1:1000. The membranes were then incubated with HRP-linked goat anti-rabbit or anti-mouse secondary antibodies (Santa Cruz) followed by visualization of immunoreactive bands by the addition of a chemiluminescent substrate (Millipore).

### Protein expression and purification

The four mammalian PDK isoforms and the E2 subunit of the PDC were amplified (PDK-1: residues 30-436; PDK-2: residues 16-407; PDK-3: residues 9-406; PDK-4: residues 1-412; E2: residues 126-233) from full length cDNA clones (Qiagen) and expressed as N-terminal His tag fusion proteins with either a TEV (PDK-1, PDK-3, PDK-4 and E2) or a Thrombin (PDK-2) protease cleavage site located between the coding sequence and the His tag. A bicistronic cDNA incorporating E1α (residues 30-390) and E1β (residues 32-359) was synthesized (DNA 2.0, CA, USA) and expressed as an N-terminal His tag fusion protein. The recombinant proteins were expressed in *E.coli* BL21 cells and purified on a Ni2+ affinity HiTrap column (GE Healthcare, USA) followed by size exclusion chromatography on a Superdex 75 column (GE Healthcare, USA) and used directly for activity and binding affinity assays. For protein crystallization, PDK-2 was treated with TEV protease to yield untagged protein. Recombinant HSP-90α and HSP-90β proteins were expressed and purified as described previously [40].

### Crystallization and X-ray structure determination

For co-crystallisation studies Human PDK-2 (16-407aa) was mixed with Compound Pfz3 (N-(2-aminoethyl)-2-{3-chloro-4-[(4-isopropylbenzyl)oxy]phenyl}acetamide); Pfizer, Patent application: EP1247860, 1-304, 2002) at 3-fold molar excess before being concentrated to 10 mg/ml. This liganded PDK-2 crystallized in 0.1 M Sodium acetate pH 5.8, 0.125 M magnesium chloride and 6% isopropanol using the hanging-drop vapour-diffusion method at 4°C. Crystals grew within 48 h.

For data collection the protein crystals were flash-frozen at 100K using a cryo-protection buffer consisting of stabilising solution and 30-35% glycerol. Diffraction data of PDK-2 in complex with VER-246608 and VER-246520 were collected at 100K on a RIGAKU RAXIS4++ image plate detector with a laboratory rotating anode generator using CuKα radiation. Data processing was carried out with the D*TREK program package [41] and data collection statistics are summarised in Supplementary Table 3. The structures were determined by molecular replacement using the published unliganded PDK-2 structure (PDBcode 2BTZ) to calculate model phases and subsequently refined using REFMAC5 [42].

Interactive graphical model building was carried out with COOT. In both structures the respective ligands were clearly defined by the initial electron density maps.

### Fluorescence polarization assay

This assay was developed using a fluorescein-labelled probe, VER-160364 (5-[({4-[5-(5-chloro-2,4-dihydroxyphenyl)-3-(ethylcarbamoyl)-1,2-oxazol-4-yl]phenyl}methyl)

carbamoyl]-2-(6-hydroxy-3-oxo-3H-xanthen-9-yl)benzoic acid), which binds to the ATP site of PDK resulting in an increase in anisotropy. PDK-1 protein was used at its Kd concentration of 30 nM, which was determined by titration with a probe concentration of 10 nM.

Compounds were tested in a 384-well format incorporating 10-point tripling titration curves in assay buffer (50 mM MOPS pH 7.5; 50 mM K_2_PO_4_;150 mM NaCl; 1 mM DTT; 5% Glycerol; 0.1% Octyl-D-β-glucopyranoside). Following a 90 min incubation period, the plates were read using a Biotek Synergy plate reader (Ex 485/20 nM; Em 528/20 nM) and Delta mP was calculated by subtracting free probe values from the values obtained at the varying compound concentrations. The data was fitted by non-linear regression using XLFIT4 within a custom ABASE (IDBS) protocol in order to determine IC_50_values. The HSP-90ß FP assay was conducted as previously described [43].

### PDK1-4 enzyme functional assay

DELFIA assay reagents (assay buffer, wash buffer, enhancement solution and anti-rabbit IgG-Eu-N1 secondary antibody) and plates were obtained from Perkin Elmer (MD, USA). Test compounds were subjected to a 10 point tripling dilution in DMSO, diluted in MOPS buffer (60mM MOPS pH7.2, 15 mM Magnesium acetate, 60 mM KCl) and added to the enzyme mix (10 nM PDK-1, 2 and 3 or 20 nM PDK-4, 300nM E1, 0.1 mg/mL BSA, 1 mM DTT) in 96-well V-bottom plates (Greiner). The reaction was initiated by the addition of ATP to a final concentration of 5 μM followed by a 1 h incubation at 30°C. The reaction was then stopped by the addition of STOP solution (50 mM Carbonate-Bicarbonate Buffer, pH 9.6), and then transferred to 96 well DEFLIA yellow plates. The plates were then sealed and incubated o/n at 4°C. Detection and quantification of p(Ser^293^)E1α levels was then achieved through incubation with anti-p(Ser^293^)E1α primary antibody (Genescript) followed by anti-rabbit secondary IgG-Eu-N1 antibody and addition of enhancement solution as per the manufacturer's instructions. The time-resolved fluorescent signal was then measured using a Victor2 plate reader (Perkin Elmer). The data was fitted by non-linear regression using XLFIT4 within a custom ABASE (IDBS) protocol in order to determine IC_50_ values.

### HSP-70 in-cell ELISA assay

DELFIA assay reagents (assay buffer, enhancement solution and europium-labeled secondary antibody) were obtained from Perkin Elmer (MD, USA). PC-3 cells were seeded (1×10^4^ per well) into 96 well black-walled cell culture plates. The following day, the cells were treated with a 10 point tripling dilution series of test compound for 24 h followed by fixation with TBS containing 4% formaldehyde for 15 min at RT. The cells were then permeabilized the addition of permeabilization buffer (TBS containing 1% triton-X-100) and a further 15 min incubation at RT. The cells were then washed 3X with TBS, followed by the addition of quenching solution (TBS containing 1% H_2_O_2_), a 20 min incubation at RT and a further 3X wash with TBS. The permeabilized cells were then incubated for 30 minutes with blocking buffer (TBS containing 5% (w/v) dried milk) followed by a 90 min incubation with HSP-70 primary antibody (ENZO; 1:1000 dilution in TBS containing 200 μg/ml BSA). The wells were then washed 3X with wash buffer (TBS containing 0.1% Tween-20), followed by the addition of europium-labeled anti-mouse secondary antibody (1:1000 in DELFIA assay buffer) and a 1 h incubation at RT. The wells were then washed 3X with wash buffer followed by the addition of enhancement solution. The time-resolved fluorescence signal was measured using a Victor2 plate reader (Perkin Elmer).

### Phospho-Serine E1α ELISA assays

Phospho(Ser^293^) E1α MSD ELISA assay: All reagents were purchased from Meso Scale Discovery (Maryland, USA) unless otherwise stated. Following compound treatment, cells were treated with lysis buffer (TBS pH 7.4, 1% Tween-20, 1 mM EGTA, 1 mM EDTA, 1 mM NaF, protease and phosphatase inhibitor cocktails (Roche)) on ice and the lysates added to standard bind 96 well MSD plates which were pre-loaded with custom phospho(Ser^293^)E1α ‘capture’ antibody (2 μg/ml in TBST containing 3% (w/v) blocker A) (Genescript) and blocked with 3% (w/v) blocker A, followed by a 1 h incubation at RT. The wells were then washed 3X with TBST (0.05% tween 20) followed by the addition of 2 μg/ml E1α ‘detection’ antibody (2 μg/ml) (Abcam) and a further 1 h incubation at RT. The wells were again washed 3X with TBST followed by the addition of 1 μg/ml Goat anti-mouse Sulfotag antibody, and a further TBST wash prior to the addition of 2X read buffer T. The chemiluminescent signal was then measured on a MSD sector imager 2400.

Cellular phospho(Ser^232^)E1α and phospho(Ser^300^)E1α levels were measured using commercially available assay kits as per the manufacturer's instructions (Abcam, UK).

### Kinase selectivity screen

A *scan*EDGE screen was performed by DiscoverRx (San Diego, CA, US) with 10 μM VER-246608 against a panel of 96 kinases encompassing the AGC, CAMK, CMGC, CK1, STE, TK, TKL, lipid, and atypical families. A proprietary active site-directed competition binding assay was employed to quantitatively measure interactions between test compounds and the individual kinases on the panel. Results were expressed as a percentage of control binding, with 0% indicating complete binding of the test compound.

### Cytotoxicity and spheroid growth assays

Compound cytotoxicity was determined using the Sulforhodamine B assay for cells cultured as a monolayer as described previously (45). For spheroid growth experiments, PC-3 cells were seeded (500 cells/well) into 96 well round bottom plates (Corning Costar 7007) in RPMI-1640 media containing 2.5% (w/v) Matrigel (BD Biosciences). The resultant spheroids were treated with VER-246608 at the indicated concentrations 48 h post-seeding. Spheroid volumes were determined by obtaining diameter measurements from images taken on a Zeiss Axiovert 200M inverted microscope using the axiovision software.

### PDC activity

Cellular PDC activity was determined using a commercial assay kit (Abcam) as per the manufacturer's instructions. Briefly, PC-3 and K562 cell suspensions at a density of 1×10^6^ and 1.5×10^6^, respectively, were transferred to 96 well plates (250 μL per well). Cells were pre-treated with compound or DMSO for 1 h in a CO_2_incubator at 37°C followed by cell lysis in 250 μL of a 9:1 dilution of assay kit detergent (containing 20 mM NaF and 40 μM of the PDK inhibitor VER-246550) for 10 min on ice. The cellular debris was then pelleted at 1000g for 10 min. A 200 μL aliquot of each lysate was then transferred to the immunocapture microplate. PDC reaction rates were determined from the slope of the reaction curves and expressed as a percentage of the vehicle (DMSO) control values.

### Oxygen consumption

Oxygen consumption was measured using an ‘oxytherm’ oxygen electrode system (Hansatech). PC-3 cells were trypsinized and re-suspended to a density of 1.7×10^6^cells/mL in RPMI-1640 media containing 10% FCS. Compounds or DMSO were then added to 1.5 mL aliquots of this suspension to yield the required final concentration followed by a 1 h incubation period in a CO_2_incubator at 37°C. The compound/vehicle treated cells were then transferred to the oxygen electrode chamber (pre-equilibrated to 37°C). Oxygen consumption was measured over a 30 minute period and the rate of oxygen consumption determined by plotting oxygen concentration against time and obtaining the slope of the line using the linear fit function within excel.

### Metabolite analysis

Pyruvate, L-lactate, D-glucose and L-alanine levels were determined using commercially available assay kits (Abcam) as per the manufacturer's instructions.

### Cell cycle and Sub-G1 analysis

PC-3 cells were seeded into 6-well plates at a density of 1×10^5^ cells per well. The following day, the media was aspirated and the cells washed with PBS followed by treatment with 20 μM VER-246608 or DMSO for 48 h in the relevant media. Following ethanol fixation and treatment with RNAse (Sigma), cellular DNA was stained with propidium iodide (50 μg/mL). The samples were then acquired on a FACSArray (BD Biosciences) flow cytometer and the data analyzed using FACSDiva software.

### Combination index (CI) analysis and potentiation assays

For drug synergy studies (CI analysis) PC-3 cells were seeded into 96 well plates and treated with a seven point doubling dilution of β-Chloro-L-alanine, VER-246608 or a fixed ratio combination of both agents. The cells were then incubated for 120 h followed by cell mass determination using the Sulforhodamine B assay. Fa (fraction affected) values were calculated by normalizing DMSO control values to 1 (a Fa value of 0.5 is equivalent to a GI_50_). Drug interactions were analyzed by the combination index (CI) method based on the method described by Chou [44]. CI values <0.9 indicates synergism, 0.9 to 1.1 additivity, and >1.1 antagonism. The data was expressed in the form of Fa-CI simulation plot generated using calcusyn software (Biosoft). Potentiation assays were carried out as described previously [45].

## SUPPLEMENTARY MATERIAL FIGURES AND TABLE



## References

[1] Warburg O (1925). Über den Stoffwechsel der Carcinomzelle. Klin Wochenschr Berl.

[2] Jadvar H, Alavi A, Gambhir SS (2009). 18F-FDG uptake in lung, breast, and colon cancers: molecular biology correlates and disease characterization. J Nucl Med.

[3] Levine AJ, Puzio-Kuter AM (2010). The control of the metabolic switch in cancers by oncogenes and tumor suppressor genes. Science.

[4] Cairns RA, Harris IS, Mak TW (2011). Regulation of cancer cell metabolism. Nat Rev Cancer.

[5] DeBerardinis RJ, Mancuso A, Daikhin E, Nissim I, Yudkoff M, Wehrli S, Thompson CB (2007). Beyond aerobic glycolysis: transformed cells can engage in glutamine metabolism that exceeds the requirement for protein and nucleotide synthesis. Proc Natl Acad Sci U S A.

[6] Bluemlein K, Glückmann M, Grüning NM, Feichtinger R, Krüger A, Wamelink M, Lehrach H, Tate S, Neureiter D, Kofler B, Ralser M (2012). Pyruvate kinase is a dosage-dependent regulator of cellular amino acid homeostasis. Oncotarget.

[7] Patel MS, Korotchkina LG (2006). Regulation of the pyruvate dehydrogenase complex. Biochem Soc Trans.

[8] Lu CW, Lin SC, Chien CW, Lin SC, Lee CT, Lin BW, Lee JC, Tsai SJ (2011). Overexpression of pyruvate dehydrogenase kinase 3 increases drug resistance and early recurrence in colon cancer. Am J Pathol.

[9] Wigfield SM, Winter SC, Giatromanolaki A, Taylor J, Koukourakis ML, Harris AL (2008). PDK-1 regulates lactate production in hypoxia and is associated with poor prognosis in head and neck squamous cancer. Br J Cancer.

[10] Koukourakis MI, Giatromanolaki A, Sivridis E, Gatter KC, Harris AL (2005). Pyruvate dehydrogenase and Pyruvate dehydrogenase kinase expression in non-small cell lung cancer and tumour-associated stroma. Neoplasia.

[11] Contractor T, Harris CR (2012). p53 negatively regulates transcription of the pyruvate dehydrogenase kinase PDK2. Cancer Res.

[12] Hsieh MC, Das D, Sambandam N, Zhang MQ, Nahlé Z (2008). Regulation of the PDK4 isozyme by the Rb-E2F1 complex. J Biol Chem.

[13] McFate T, Mohyekdin A, Lu H, Thakar J, Henriques J, Halim ND, Schell MJ, Tsang TM, Teahan O, Zhou S, Califano JA, Jeoung NH, Harris RA, Verma A (2008). Pyruvate dehydrogenase complex activity controls metabolic and malignant phenotype in cancer cells. J Biol Chem.

[14] Kim JW, Tchernyshyov I, Semenza G, Dang CV (2006). HIF-1-mediated expression of pyruvate dehydrogenase kinase: a metabolic switch required for cellular adaptation to hypoxia. Cell Metab.

[15] Papandreou I, Cairns RA, Fontana L, Lim AL, Denko NC (2006). HIF-1 mediates adaptation to hypoxia by actively downregulating mitochondrial oxygen consumption. Cell Metabolism.

[16] Bonnet S, Archer SL, Allalunis-Turner J, Haromy A, Beaulieu C, Thompson R, Lee CT, Lopaschuk GD, Puttagunta L, Bonnet S, Harry G, Hashimoto K, Porter CJ, Andrade MA, Thebaud B, Michelakis ED (2007). A mitochondrial-K+ channel axis is suppressed in cancer and its normalization promotes apoptosis and inhibits cancer growth. Cancer Cell.

[17] Stockwin LH, Yu SX, Borgel S, Hancock C, Wolfe TL, Phillips LR, Hollingshead MG, Newton DL (2010). Sodium dichloroacetate selectively targets cells with defects in the mitochondrial ETC. Int J Cancer.

[18] Wong JY, Huggins GS, Debidda M, Munshi NC, De Vivo I (2008). Dichloroacetate induces apoptosis in endometrial cancer cells. Gynecologic Oncolog.

[19] Sun RC, Fadia M, Dahlstrom JE, Parish CR, Board PG, Blackburn AC (2010). Reversal of the glycolytic phenotype by dichloroacetate inhibits metastatic breast cancer cell growth *in vitro* and *in vivo*. Breast Can Res Treat.

[20] Tzeng HF, Blackburn AC, Board PG, Anders MW (2000). Polymorphism- and species-dependent inactivation of glutathione transferase zeta by dichloroacetate. Chem Res Toxicol.

[21] Hiromassa Y, Yan X, Roche TE (2008). Specific ion influences on self-association of pyruvate dehydrogenase kinase isoform 2 (PDHK2), binding of PDHK2 to the L2 lipoyl domain, and effects of the lipoyl group-binding site inhibitor, Nov3r. Biochemistry.

[22] Mazurek S, Michel A, Eigenbrodt E (2007). Effect of extracellular AMP on cell proliferation and metabolism of breast cancer cell lines with high and low glycolytic rates. J Biol Chem.

[23] Wise DR, Thompson CB (2010). Glutamine addiction: a new therapeutic target in cancer. Trends Biochem Sci.

[24] Baracca A, Chiaradonna F, Sgarbi G, Soliani G, Alberghina L, Lenaz G (2010). Mitochondrial Complex I decrease is responsible for bioenergetic dysfunction in K-ras transformed cells. Biochimica et Biophysica Acta.

[25] Gaglio D, Metallo CM, Gameiro PA, Hiller K, Danna LS, Balestrieri C (2011). Oncogenic K-Ras decouples glucose and glutamine metabolism to support cancer cell growth. Molecular Systems Biology.

[26] Heiden MG (2009). Understanding the Warburg effect: the metabolic requirements of cell proliferation. Science.

[27] Vander Heiden MG (2011). Targeting cancer metabolism: a therapeutic window opens. Nat Rev Drug Discov.

[28] Kaplon J, Zheng L, Meissl K, Chaneton B, Selivanov VA, Mackay G, van der Burg SH, Verdegaal EM, Cascante M, Shlomi T, Gottlieb E, Peeper DS (2013). A key role for mitochondrial gatekeeper pyruvate dehydrogenase in oncogene-induced senescence. Nature.

[29] Hitosugi T, Fan J, Chung TW, Lythgoe K, Wang X, Xie J, Ge Q, Gu TL, Polakiewicz RD, Roesel JL, Chen GZ, Boggon TJ, Lonial S, Fu H, Khuri FR, Kang S, Chen J (2011). Tyrosine phosphorylation of mitochondrial pyruvate dehydrogenase kinase 1 is important for cancer metabolism. Mol Cell.

[30] Mayers RM, Butlin RJ, Kilgour E, Leighton B, Martin D, Myatt J, Orme JP, Holloway BR (2003). AZD7545, a novel inhibitor of pyruvate dehydrogenase kinase 2 (PDHK2), activates pyruvate dehydrogenase *in vivo* and improves blood glucose control in obese (fa/fa) Zucker rats. Biochem Soc Trans.

[31] Aicher TD, Anderson RC, Gao J, Shetty SS, Coppola GM, Stanton JL, Knorr DC, Sperbeck DM, Brand LJ, Vinluan CC, Kaplan EL, Dragland CJ, Tomaselli HC (2000). Secondary amides of (R)-3,3,3-trifluoro-2-hydroxy-2-methylpropionic acid as inhibitors of pyruvate dehydrogenase kinase. J Med Chem.

[32] Wynn RM, Kato M, Chuang JL (2008). , Tso, SC, Li, J, Chuang, DT. Pyruvate dehydrogenase kinase-4 structures reveal a metastable open conformation fostering robust core-free basal activity. J Biol Chem.

[33] Patel MS, Korotchkina LG (2006). Regulation of the pyruvate dehydrogenase complex. Biochem Soc Trans.

[34] Miao P, Sheng S, Sun X, Liu J, Huang G (2013). Lactate dehydrogenase A in cancer: a promising target for diagnosis and therapy. IUBMB Life.

[35] Heshe D, Hoogestraat S, Brauckmann C, Karst U, Boos J, Lanvers-Kaminsky C (2011). Dichloroacetate metabolically targeted therapy defeats cytotoxicity of standard anticancer drugs. Cancer Chemother Pharmacol.

[36] Morani F, Phadngam S, Follo C, Titone R, Thongrakard V, Galetto A, Alabiso O, Isidoro C (2014). PTEN deficiency and mutant p53 confer glucose-addiction to thyroid cancer cells: impact of glucose depletion on cell proliferation, cell survival, autophagy and cell migration. Genes Cancer.

[37] Calorini L, Peppicelli S, Bianchini F (2012). Extracellular acidity as favouring factor of tumor progression and metastatic dissemination. Exp Oncol.

[38] Martinez-Outschoorn UE, Pavlides S, Howell A, Pestell RG, Tanowitz HB, Sotgia F, Lisanti MP (2011). Stromal-epithelial metabolic coupling in cancer: integrating autophagy and metabolism in the tumor microenvironment. Int J Biochem Cell Biol.

[39] Sonveaux P, Végran F, Schroeder T, Wergin MC, Verrax J, Rabbani ZN, De Saedeleer CJ, Kennedy KM, Diepart C, Jordan BF, Kelley MJ, Gallez B, Wahl ML, Feron O, Dewhirst MW (2008). Targeting lactate-fueled respiration selectively kills hypoxic tumor cells in mice. J Clin Invest.

[40] Brough PA, Aherne W, Barril X, Borgognoni J, Boxall K, Cansfield JE, Cheung KM, Collins I, Davies NG, Drysdale MJ, Dymock B, Eccles SA, Finch H (2008). 4,5-diarylisoxazole Hsp90 chaperone inhibitors: potential therapeutic agents for the treatment of cancer. J Med Chem.

[41] Pflugrath JW (1999). The finer things in X-ray diffraction data collection. Acta Crystallogr.

[42] Murshudov GN, Vagin AA, Dodson EJ (1997). Refinement of Macromolecular Structures by the Maximum-Likelihood Method. Acta Cryst.

[43] Schilb A, Riou V, Schoepfer Ottl J, Müller K, Chene P, Mayr LM, Filipuzzi I (2004). Development and implementation of a highly miniaturized confocal 2D-FIDA-based high-throughput screening assay to search for active site modulators of the human heat shock protein 90β. J Biomol Screen.

[44] Chou TC, Chou TC (1991). The median-effect principle and the combination index for quantitation of synergism and antagonism. Synergism and antagonism in chemotherapy.

[45] Massey AJ, Borgognoni J, Bentley C, Foloppe N, Fiumana A, Walmsley L (2010 18). Context-dependent cell cycle checkpoint abrogation by a novel kinase inhibitor. PLoS One.

