# Supplementary material for: VER-246608, a novel pan-isoform ATP competitive inhibitor of pyruvate dehydrogenase kinase, disrupts Warburg metabolism and induces context-dependent cytostasis in cancer cells
Source: Oncotarget. 2014 Nov 2;5(24):12862–76. doi: 10.18632/oncotarget.2656 (PMC4350332; doi:10.18632/oncotarget.2656)
Supplement: Supplementary file 1 [file oncotarget-05-12862-s001.pdf]

VER-246608, a novel pan-isoform ATP competitive inhibitor of pyruvate dehydrogenase kinase, disrupts warburg metabolism and induces context-dependent cytostasis in cancer cells

Supplementary Material

^{13}C-NMR metabolite analysis

Jurkat cells (5 X 10^6) were pre-treated in RPMI-1640 media containing either DMSO or 20 μM VER-00246608 for 1 h. The cells were then pelleted and washed in PBS containing DMSO/compound and re-suspended in glucose-free RPMI-1640 media containing 10% FCS supplemented with 2 g/L 1,6-^{13}C-D-Glucose and either DMSO or 20 μM VER-246608. The cell suspension was then transferred to a 5mm NMR tube and placed into the sample probe of a Bruker DRX600 NMR spectrometer. The probe was pre-equilibrated to 37°C and shimmed on a matched sample. Pulses were also calibrated on the matched sample. The sample containing the Jurkat cells was rapidly shimmed and data acquisition initiated within 15 minutes.

1D ^{13}C NMR spectra (240ppm sweep width, 64k acquired points, 64 scans) were then acquired at 10 minute intervals up to 2 h. The glucose and lactate peaks were converted to concentrations by referencing to a cell-free reference sample containing the same concentration of 1,6-^{13}C-D-Glucose (11 mM).

Synthesis of VER-246608 and VER-246520

<u>Scheme 1.</u> Synthesis of VER-246608

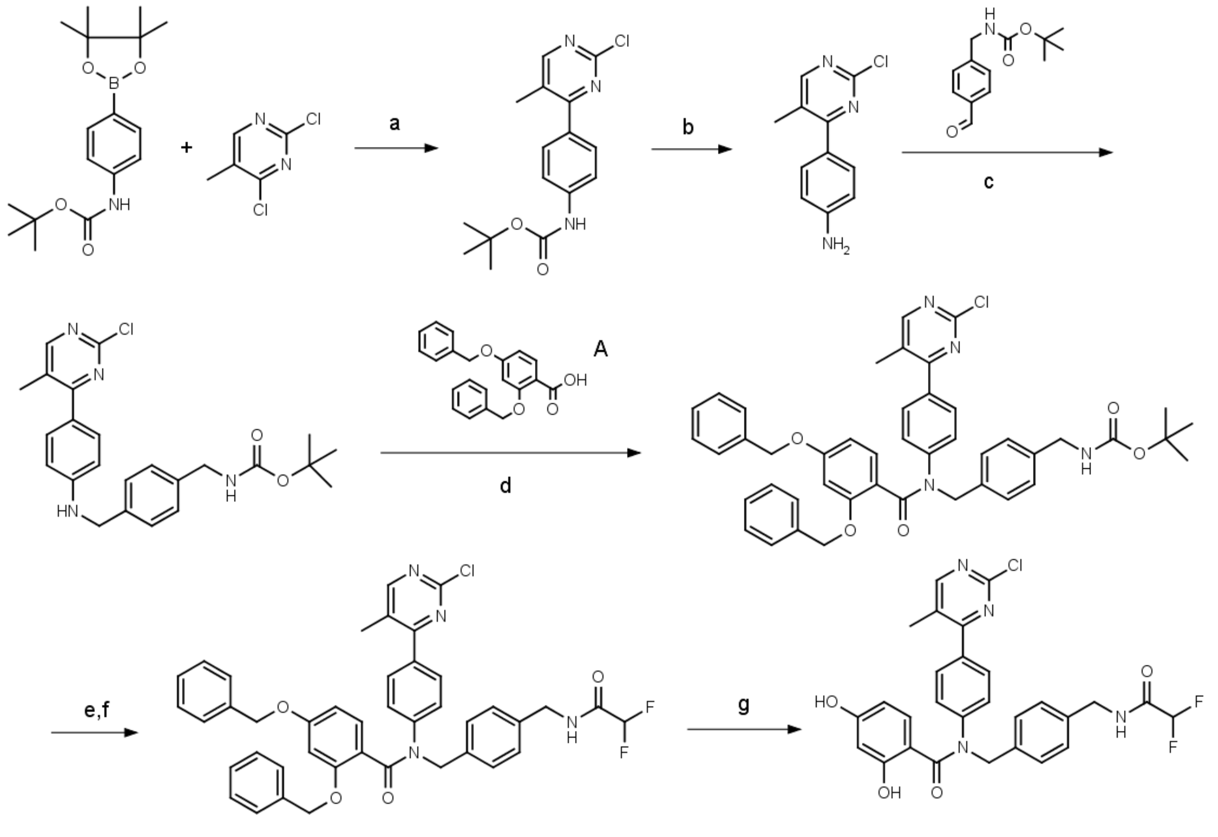

Reagents and Conditions: (a) K_2CO_3, $PdCl_2$(*dppf*)$_2$, THF, H_2O, 60°C, 16 hr; (b) TFA, DCM, rt, 16 hr; (c) $NaBH(OAc)_3$, AcOH, DCM, rt, 16 hr; (d) (*i*) **A**, $(COCl)_2$, DCM, cat. DMF, rt, 2 hr (*ii*), Et_3N, DCM, 16 hr; (e) TFA, DCM, rt, 4 hr; (f) CF_2HCO_2H, $Et^{i}Pr_2NH$, MeCN, 80°C, 1 hr; (g) BCl_3, DCM, -78°C to rt, 4.5 hr.

General Procedures

All reagents obtained from commercial sources were used without further purification. Anhydrous solvents were obtained from commercial sources and used without further drying. Flash chromatography was performed with pre-packed silica gel cartridges (Strata SI-1; 61Å, Phenomenex, Cheshire UK or IST Flash II, 54Å, Argonaut, Hengoed, UK) or by automated flash chromatography using a Combi*Flash* R_f apparatus (Teledyne Isco Inc.) using Redi*Sep* R_f pre-packed silica columns (Teledyne Isco Inc.) or Sila*Sep* pre-packed columns (Silicycle

Inc.). Thin layer chromatography was conducted with 5 x 10 cm plates coated with Merck Type 60 F_{254} silica gel. The compounds were characterized by high performance liquid chromatography-mass spectroscopy (HPLC-MS) on either an Agilent HP1200 Rapid Resolution Mass detector 6140 multimode source M/z range 150 to 1000 amu or an Agilent HP1100 Mass detector 1946D ESI source M/z range 150 to 1000 amu. The conditions and methods listed below are identical for both machines.

Column for 3.75 min run: GeminiNX, 5µm, C18, 30 x 2.1mm (Phenomenex) or Zorbax Eclipse Plus, 3.5µm, C18, 30 x 2.1mm (Agilent).Temperature: 35°C.

Mobile Phases:

A - H_2O + 10 mmol / ammonium formate + 0.08% (v/v) formic acid at pH ca 3.5.

B - 95% Acetonitrile + 5% A + 0.08% (v/v) formic acid.

Injection Volume: 1µL

Method A “Short” method gradient table, either positive (pos) or positive and negative (pos/neg) ionization

Time (min)	Solvent A (%)	Solvent B (%)	Flow (mL/min)
0	95	5	1
0.25	95	5	1
2.50	5	95	1
2.55	5	95	1.7
3.60	5	95	1.7
3.65	5	95	1
3.70	95	5	1
3.75	95	5	1

Method B “Super Short” method gradient table, either positive (pos) or positive and negative (pos / neg) ionisation

Time (min)	Solvent A (%)	Solvent B (%)	Flow (mL/min)
0	95	5	1.3
0.12	95	5	1.3
1.30	5	95	1.3
1.35	5	95	1.6
1.85	5	95	1.6
1.90	5	95	1.3
1.95	95	5	1.3

Nuclear magnetic resonance (NMR) analysis was performed with a Brucker DPX-400 MHz NMR spectrometer. The spectral reference was the known chemical shift of the solvent. Proton NMR data is reported as follows: chemical shift (□) in ppm, multiplicity (s = singlet, d = doublet, t = triplet, q = quartet, p = pentet, m = multiplet, dd = doublet of doublet, br = broad), integration, coupling constant.

Preparative HPLC purifications were performed on a Waters FractionLynx MS Autopurification system with a Gemini® 5 μM C18(2), 100 mm × 20 mm i.d. column from Phenomenex, running at a flow rate of 20 mL min^{-1} with UV diode array detection (210 – 400 nm) and mass-directed collection.

At pH 4:

Solvent A: HPLC grade Water + 10mM ammonium acetate + 0.08% v/v formic acid.

Solvent B: 95% v/v HPLC grade acetonitrile + 5% v/v Solvent A + 0.08% v/v formic acid.

Typical gradient: 5 to 95% B over 9.5 minutes.

2,4-bis(benzyloxy)benzoic acid (intermediate A)

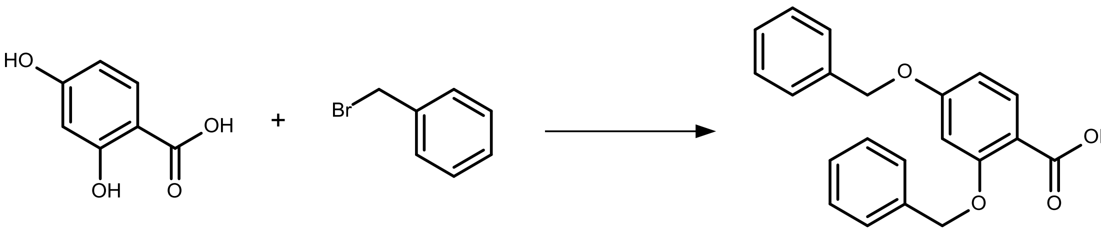

2,4-dihydroxybenzoic acid (Aldrich, 29.3 g, 0.19 mol) was stirred in DMF (500 mL) and potassium carbonate (105 g, 0.76 mol) was added. To this stirred mixture was added a solution of benzyl bromide (99.2 g, 69 mL, 0.58 mol) in DMF (100 mL) drop-wise and the reaction was then stirred at ambient temperature overnight. A further 7 mL of benzyl bromide was added and the reaction mixture heated at 50°C for 2 hours to give complete conversion to the tris-benzylated intermediate product. When benzylation was complete (by LCMS analysis), an aqueous solution of potassium hydroxide (17 g, 0.3 mol) in water (200 mL) was added followed by methanol (300 mL) and the mixture heated at reflux until complete hydrolysis of the benzyl ester was observed. When reaction was complete, the mixture was allowed to cool to ambient temperature and poured carefully into a stirred solution of 1N HCl (2000 mL) and then extracted with ethyl acetate (4 x 500mL). The combined extracts were washed with brine (5 x 800 mL), water (800 mL), dried ($MgSO_4$) and concentrated *in vacuo*. The resultant solid material was washed with petroleum ether and diethyl ether and dried *in vacuo* to yield 2,4-*bis*(benzyloxy)benzoic acid (intermediate A) (63.53 g, 96%) as an off white solid.

LC/MS (method A): RT = 2.63 min; *m/z* = 333 $[M-H]^-$. Total run time 3.75 mins.

Step 1

tert-butyl-N-[4-(2-chloro-5-methylpyrimidin-4-yl)phenyl]carbamate

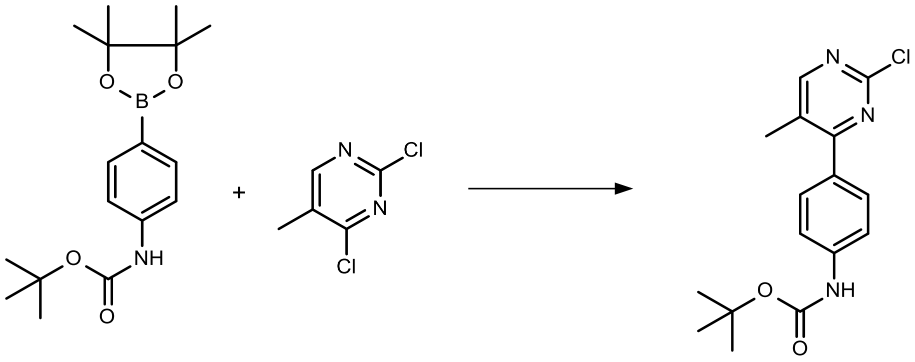

A mixture of *tert*-butyl N-[4-(tetramethyl-1,3,2-dioxaborolan-2-yl)phenyl]carbamate (Fluorochem, 6.00 g, 18.8 mmol), 2,4-dichloro-5-methylpyrimidine (Aldrich, 3.83 g, 23.50 mmol) and potassium carbonate (7.79 g, 56.4 mmol) in THF (90 mL) and water (10 mL) was degassed by bubbling nitrogen gas through the mixture for 10 minutes. [1,1'-*bis*(diphenylphosphino)ferrocene]dichloropalladium (II) (687 mg, 0.94 mmol) was added and the mixture was heated at 60°C overnight under a nitrogen atmosphere. The reaction mixture was allowed to cool to ambient temperature and partitioned between ethyl acetate (200 mL) and water (200 mL). The phases were separated and the organic phase was dried ($MgSO_4$), filtered and filtrate solvents removed *in vacuo* to afford crude product as an oil which was purified by flash column chromatography on silica gel, eluting with a gradient of hexane to 50% hexane in EtOAc to afford *tert*-butyl N-{[4-(2-chloro-5-methylpyrimidin-4-yl)phenyl]methyl}carbamate as a pale yellow gum (6.63 g).

LC/MS (method A): RT = 2.59 min; *m/z* = 320 $[M+H]^+$. Total run time 3.75 mins.

Step 2

4-(2-chloro-5-methylpyrimidin-4-yl)aniline

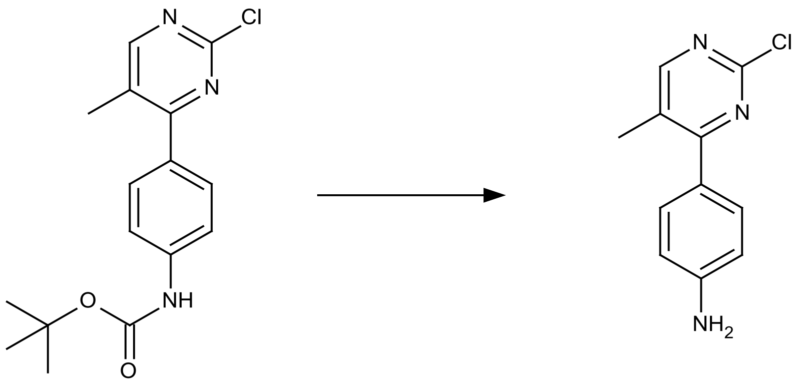

To a stirred solution of *tert*-butyl-N-[4-(2-chloro-5-methylpyrimidin-4-yl)phenyl] carbamate (6.01 g, 18.8 mmol) in DCM (100 mL) at room temperature was added trifluoroacetic acid (7.20 mL, 94 mmol). The resulting mixture was left standing at room temperature for 16 h. The reaction mixture was evaporated below 50°C and then co-evaporated *in vacuo* with toluene (3 x 20 mL) to give the [4-(2-chloro-5-methylpyrimidin-4-yl)phenyl]methanamine as the trifluoroacetic acid salt (8.3 g), which was used without further purification.

LC/MS (method A): RT = 2.04 min; *m/z* = 220 $[M+H]^{+}$. Total run time 3.75 mins.

Step 3

tert-butyl-N-{[4-({[4-(2-chloro-5-methylpyrimidin-4-yl)phenyl]amino}methyl) phenyl]methyl}carbamate

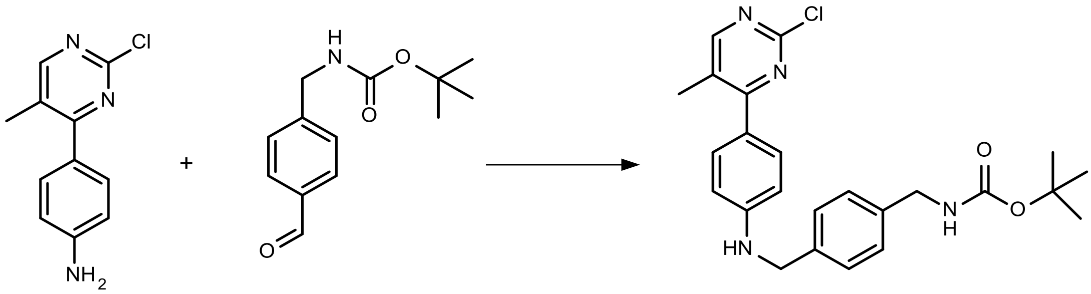

tert-butyl N-[(4-formylphenyl)methyl]carbamate (Maybridge UK, 1.00 g, 4.25 mmol) and 4-(2-chloro-5-methylpyrimidin-4-yl)aniline (TFA salt, 1.42 g, 4.25 mmol) were dissolved in DCM (100 mL) and sodium triacetoxyborohydride (0.99 g, 4.68 mmol) was added followed by acetic acid (0.24 mL, 4.25 mmol). The reaction mixture was stirred at RT for 16 hr, then cooled to 0°C and NaOH solution (1.0M, 100 mL) was added and the resulting phases were

separated. The aqueous phase was re-extracted with DCM (3 x 20 mL) and the combined organic phases were washed with sat NaCl (aq), dried over $MgSO_4$ and filtered. The filtrate solvents were removed *in vacuo* to afford the crude product as an oil which was purified by flash column chromatography on silica gel, eluting with a gradient of 0 to 100% hexane in EtOAc to afford *tert*-butyl-N-{[4-({[4-(2-chloro-5-methylpyrimidin-4-yl)phenyl]amino}methyl) nphenyl]methyl}carbamate as a yellow gum (1.8 g, 96%).

LC/MS (method A): RT = 2.71 min; *m/z* = 383 $[M\text{-}t\text{-}Butyl+H]^+$. Total run time 3.75 mins.

Step 4

tert-butyl-N-{[4-({1-[2,4-bis(benzyloxy)phenyl]-N-[4-(2-chloro-5-methylpyrimidin-4-yl)phenyl]formamido}methyl)phenyl]methyl}carbamate

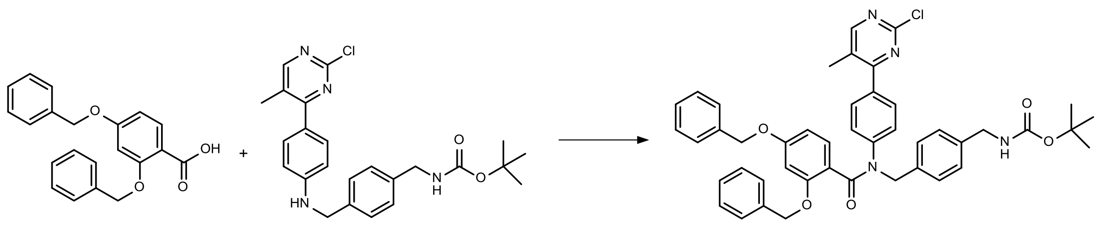

Oxalyl chloride (2.0M in DCM, 10.65 mL, 21.3 mmol) was added to a stirred solution of 2,4-*bis*(benzyloxy)benzoic acid ([**A**], 4.27 g, 12.78 mmol) in DCM (100 mL). Two drops of DMF were added and the reaction was stirred at RT for ca 2 hour then the solvents removed *in vacuo*. The resultant residue was dissolved in DCM (100 mL) then triethylamine (3 mL, 21.30 mmol) was added followed by *tert*-butyl-N-{[4-({[4-(2-chloro-5-methylpyrimidin-4-yl)phenyl]amino}methyl)phenyl]methyl}carbamate (1.87 g, 4.26 mmol). The reaction mixture was stirred at RT overnight, then diluted with DCM and washed with sat. aq. $NaHCO_3$ solution then sat. NaCl (aq). The organic extract was dried ($MgSO_4$) then the solvent removed *in vacuo* and the crude product purified by flash column chromatography on silica gel eluting with 0 to 100% EtOAc in hexane to afford product as a yellow foam (2.04 g, 64%).

LC/MS (method A): RT = 1.50 min; *m/z* = 699 $[M\text{-}t\text{-}Butyl+H]^+$. Total run time 3.75 mins.

Step 5

N-{[4-(aminomethyl)phenyl]methyl}-2,4-bis(benzyloxy)-N-[4-(2-chloro-5-methylpyrimidin-4-yl)phenyl]benzamide

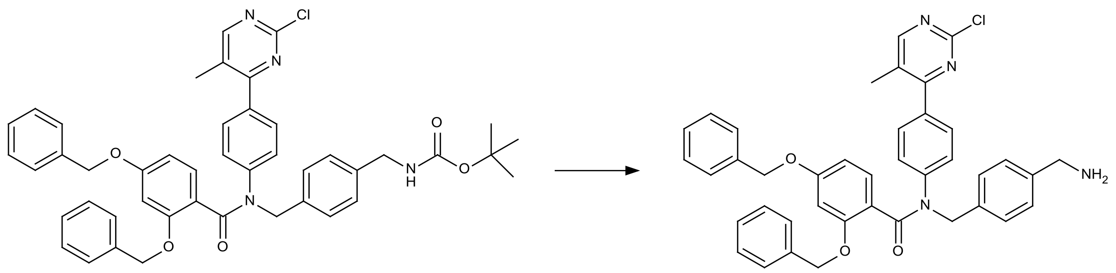

Trifluoroacetic acid (0.27 mL) was added drop-wise to a stirred solution of tert-butyl-N-{[4-({1-[2,4-bis(benzyloxy)phenyl]-N-[4-(2-chloro-5-methylpyrimidin-4-yl)phenyl] formamido}methyl)phenyl]methyl}carbamate (264 mg, 0.35 mmol) in anhydrous DCM (2 mL) under a nitrogen atmosphere and stirred for 4 hours. The reaction mixture was evaporated *in vacuo* and then DCM was added (30 mL). The solution was washed with sat $NaHCO_3$ solution (2 x 30 mL), sat. NaCl (aq) solution (1 x 30 mL), then dried over $MgSO_4$. The mixture was filtered and the filtrate evaporated to a brown oil which was dried under high vacuum to afford 174 mg (76%) of product which was used without further purification.

Step 6

2,4-bis(benzyloxy)-N-[4-(2-chloro-5-methylpyrimidin-4-yl)phenyl]-N-({4-[(2,2-difluoroacetamido)methyl]phenyl}methyl)benzamide

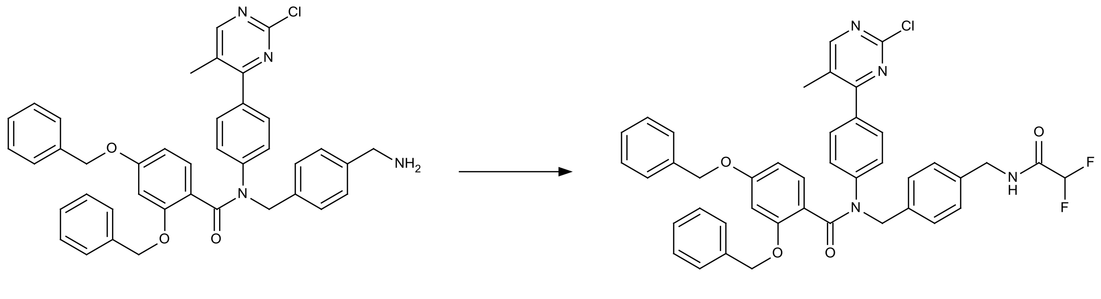

N-{[4-(aminomethyl)phenyl]methyl}-2,4-bis(benzyloxy)-N-[4-(2-chloro-5-methylpyrimidin-4-yl)phenyl]benzamide (90 mg, 0.12 mmol) and O-(7-azabenzotriazol-1-yl)-*N,N,N',N'*-tetramethyluronium hexafluorophosphate (HATU) (91 mg, 0.12 mmol) were dissolved in

acetonitrile (2 mL) in a microwave vial. Difluoroacetic acid (11.2 mg, 0.12 mmol) was added followed by diisopropylethylamine (0.08 mL, 0.48 mmol). The mixture was heated in a microwave synthesiser at 80°C for 1 hour then allowed to cool. Solvents were removed *in vacuo* and the solid was purified by flash column chromatography on silica gel eluting with 0 to 100% EtOAc in hexane to afford product as an off-white gum (46 mg, 56%).

LC/MS (method A): RT = 2.80 min; *m/z* = 733 $[M+H]^+$. Total run time 3.75 mins.

Step 7

N-[4-(2-chloro-5-methylpyrimidin-4-yl)phenyl]-N-({4-[(2,2-difluoroacetamido) methyl]phenyl}methyl)-2,4-dihydroxybenzamide (VER-246608)

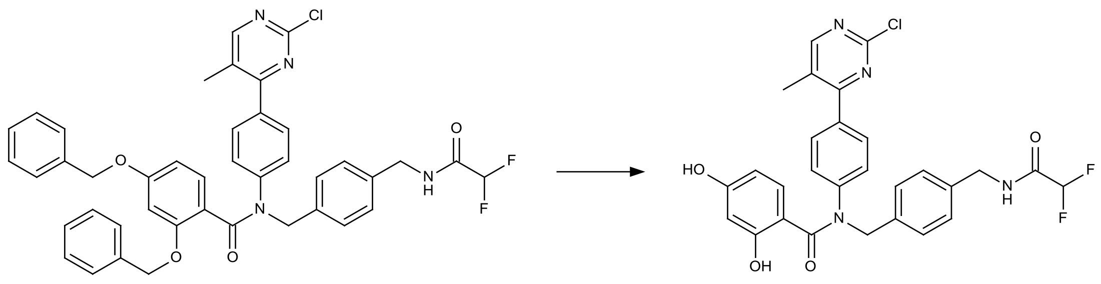

2,4-bis(benzyloxy)-N-[4-(2-chloro-5-methylpyrimidin-4-yl)phenyl]-N-({4-[(2,2-difluoro acetamido)methyl]phenyl}methyl)benzamide (46 mg, 0.06 mmol) was dissolved in anhydrous DCM (10 mL) and the mixture was cooled under a nitrogen atmosphere to -78°C with solid CO_2 acetone bath. Boron trichloride solution was added (1.0M in DCM, 0.3 mL, 0.3 mmol) and the mixture stirred for 30 mins at -78°C and then for 4 hours at RT. The mixture was re-cooled to -78°C and quenched with methanol. The reaction warmed to RT then evaporated *in vacuo* to give a crude solid which was purified by flash column chromatography on silica gel eluting with 0 to 10% MeOH in DCM to afford product as an off-white solid. Further purification by preparative HPLC at pH = 4 afforded N-[4-(2-chloro-5-methylpyrimidin-4-yl)phenyl]-N-({4-[(2,2-difluoroacetamido)methyl]phenyl}methyl)-2,4-dihydroxybenzamide as a colourless solid (6 mg, 18%).

LC/MS (method A): RT = 2.33 min; *m/z* = 553 $[M+H]^+$. Total run time 1.95 mins.

^{1}H NMR (d_6 DMSO): □ 2.27 (s, 3H), 4.30 (d, 2H, *J* = 6.1 Hz), 5.10 (s, 2H), 6.09 (dd, 1H, *J* = 8.3, 2.3 Hz), 6.12 (d, 1H, *J* = 2.3 Hz), 6.26 (t, 1H, J_{H-F} = 53.5 Hz), 6.93 (d, 1H, *J* = 8.3 Hz), 7.19 – 7.23 (m, 4H), 7.31 (d, 2H, *J* = 8.0 Hz), 7.49 (d, 2H, *J* = 8.8 Hz), 8.65 (s, 1H), 9.30 (t, 1H, *J* = 6.0Hz), 9.49 – 9.79 (brs, 1H), 9.81-10.02 (brs, 1H).

Scheme 2. Synthesis of VER-246520

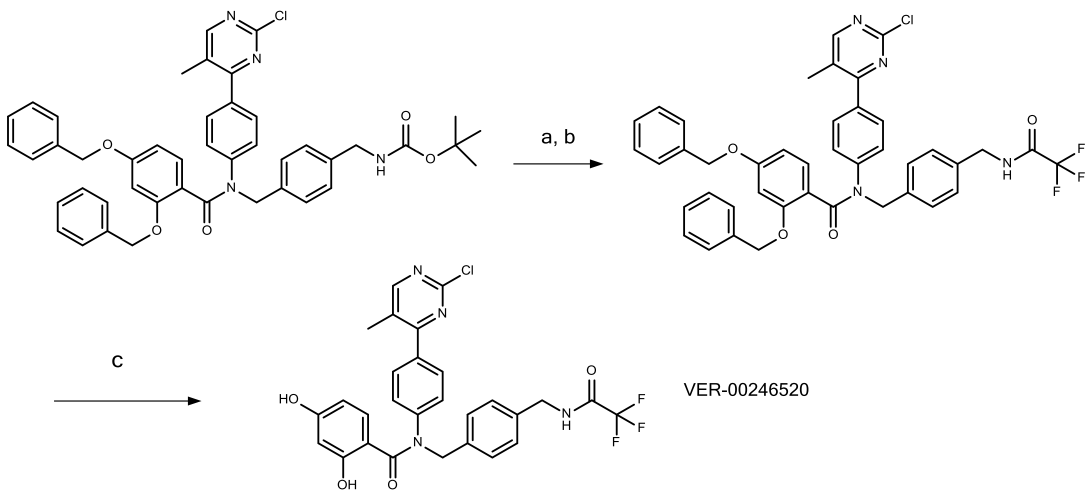

Reagents and Conditions: (a) TFA, DCM, rt, 16 hr; (b) Et_3N, DCM, TFAA, rt, 4 hr; (c) BCl_3, DCM, -78°C to rt, 4 hr.

Step 1

2,4-bis(benzyloxy)-N-[4-(2-chloro-5-methylpyrimidin-4-yl)phenyl]-N-({4-[(trifluoroacetamido)methyl]phenyl}methyl)benzamide

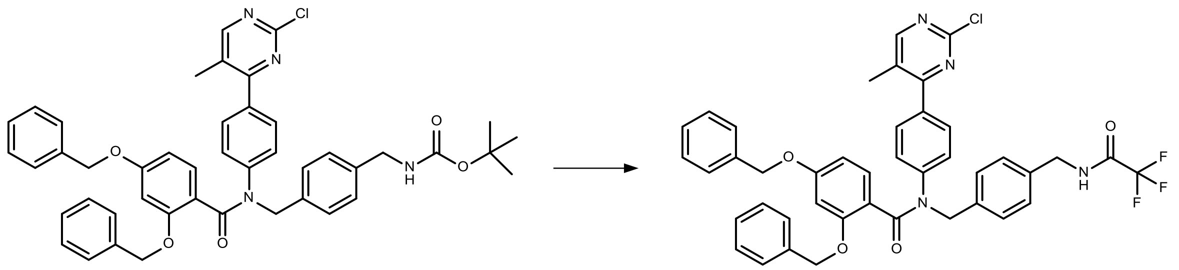

Trifluoroacetic acid (0.33 mL, 4.37 mmol, 10 equiv) was added drop-wise to a solution of tert-butyl-N-{[4-({1-[2,4-bis(benzyloxy)phenyl]-N-[4-(2-chloro-5-methylpyrimidin-4-yl)phenyl]formamido}methyl)phenyl]methyl}carbamate (330 mg, 0.437 mmol) in anhydrous DCM (5 mL) under a nitrogen atmosphere and the reaction mixture was stirred at ambient temperature for 16 hours. The solvent and excess TFA was removed *in vacuo* and then the residue re-dissolved in anhydrous DCM (5 mL) under nitrogen atmosphere. Triethylamine (0.30 mL, 5 equiv) was added followed by drop-wise addition of trifluoroacetic anhydride (0.09 mL, 1.5 equiv). The reaction mixture was stirred at ambient temperature for 4 hours. The reaction mixture was concentrated *in vacuo* and the residue partitioned between EtOAc (50 mL) and water (50 mL). The phases were separated and the organic phase was washed with sat. NaCl solution (50 mL), dried over $MgSO_4$, filtered and the filtrate solvents evaporated to leave a yellow oil which was adsorbed onto silica gel then purified by automated flash chromatography (Combi*Flash R*f, 12g Sila*Sep* silica column), eluting with 0-100% EtOAc in Hexane to afford 2,4-bis(benzyloxy)-N-[4-(2-chloro-5-methylpyrimidin-4-yl)phenyl]-N-({4[(trifluoroacetamido)methyl] phenyl} methyl) benzamide as an off-white foam (233 mg, 71%).

Step 2

N-[4-(2-chloro-5-methylpyrimidin-4-yl)phenyl]-2,4-dihydroxy-N-({4-[(trifluoroacetamido)methyl]phenyl}methyl)benzamide (VER-246520)

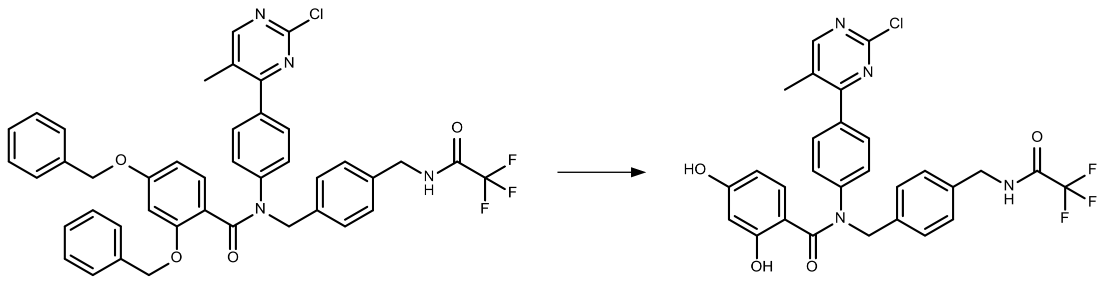

To a solution of 2,4-bis(benzyloxy)-N-[4-(2-chloro-5-methylpyrimidin-4-yl)phenyl]-N-({4-[(trifluoroacetamido)methyl]phenyl}methyl)benzamide (231 mg, 0.31 mmol) in anhydrous DCM (7 mL), cooled with ice water bath, was added drop-wise a solution of boron trichloride (1.0M in DCM 1.54 mL, 1.54 mmol). A precipitate forms during the addition and the resulting mixture was stirred at 0°C for 10 mins, then cooling bath was removed and mixture stirred at ambient temperature for 4 hours. LCMS analysis showed no s/m remains and a major product (>90%) with mass consistent with stated product. The reaction mixture was cooled with an ice-water bath and then quenched by drop-wise addition of methanol (5 mL). The solvents were removed *in vacuo* and the residue was partitioned between EtOAc (30 mL) and water (30 mL). The phases were separated and the organic phase was washed with brine (50 mL), dried over $MgSO_4$ and filtered the filtrate solvent was removed *in vacuo* to afford a light-brown oil. The crude product was purified by automated flash chromatography, eluting with 0 to 100% EtOAc in hexane (CombiFlash Rf, 12g SilaSep™ column) to afford the product as a viscous oil which slowly transformed to a colorless glass, The product was isolated by trituration with diethyl ether/hexane mix, filtered and dried *in vacuo* to afford N-[4-(2-chloro-5-methylpyrimidin-4-yl)phenyl]-2,4-dihydroxy-N-({4-[(trifluoroacetamido)methyl]phenyl}methyl)benzamide (VER-246520) as a colourless solid, 74 mg, 42%, (solvated 0.13 with diethyl ether).

LC/MS (method B): RT = 1.29 min; *m/z* = 571 $[M+H]^+$. Total run time 3.75 mins.

^{1}H NMR (399 MHz, DMSO-d_6) δ 9.96 (s, 1H), 9.89 (s, 1H), 9.58 (s, 1H), 8.66 (s, 1H), 7.50 (d, 2H), 7.34 (d, *J* = 7.97 Hz, 2H), 7.26 – 7.18 (m, 4H), 6.96 (d, *J* = 8.14 Hz, 1H), 6.16 – 6.05 (m, 2H), 5.11 (s, 2H), 4.36 (d, *J* = 5.18 Hz, 2H), 2.25 (s, 3H).

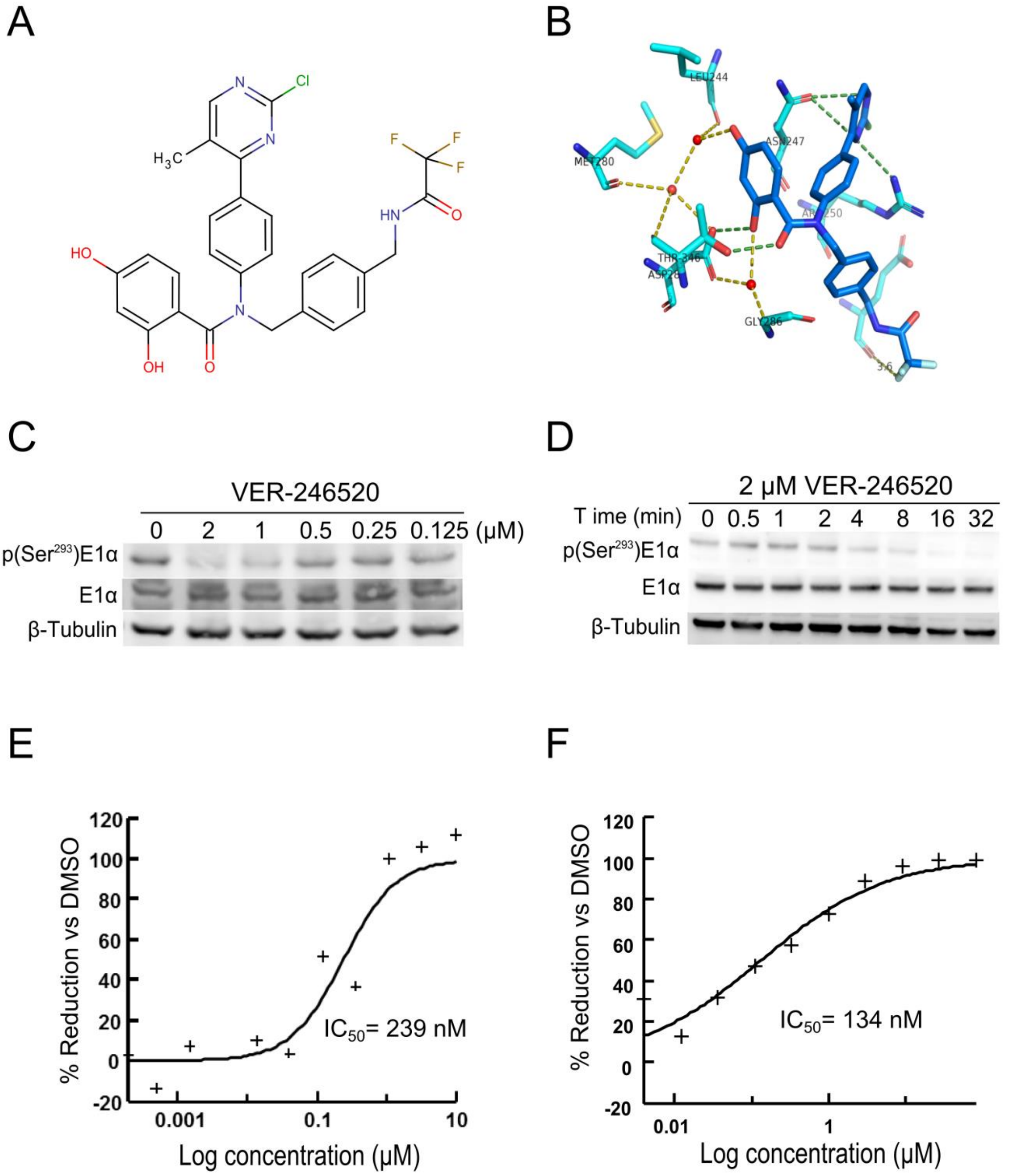

Supplementary Figure S1: VER-246520 is potent ATP-competitive inhibitor of PDK. A, molecular structure of VER-246520 (MW, 552.96 KDa). B, X-ray crystal structure (2.5 Ǻ) of VER-246520 in the ATP binding site of PDK-2. Water molecules are represented as red spheres and dashed lines indicate hydrogen bonds. C and D, PC-3 cells were seeded into 60mm culture dishes and treated with the indicated concentrations of VER-246520 or DMSO control for 90 minutes (C) or for varying time periods (D). Cell lysates were then subjected to immunoblot analysis with antibodies against the indicated proteins. β-tubulin served as a loading control. E, representative DELFIA functional assay IC_{50} curve illustrating the concentration dependent inhibition of PDK-1 enzyme activity by VER-246520. F, representative fluorescence polarization assay binding curve demonstrating the affinity of VER-246520 for PDK-1 through competitive binding with a fluorescently labelled ATP site binding probe.

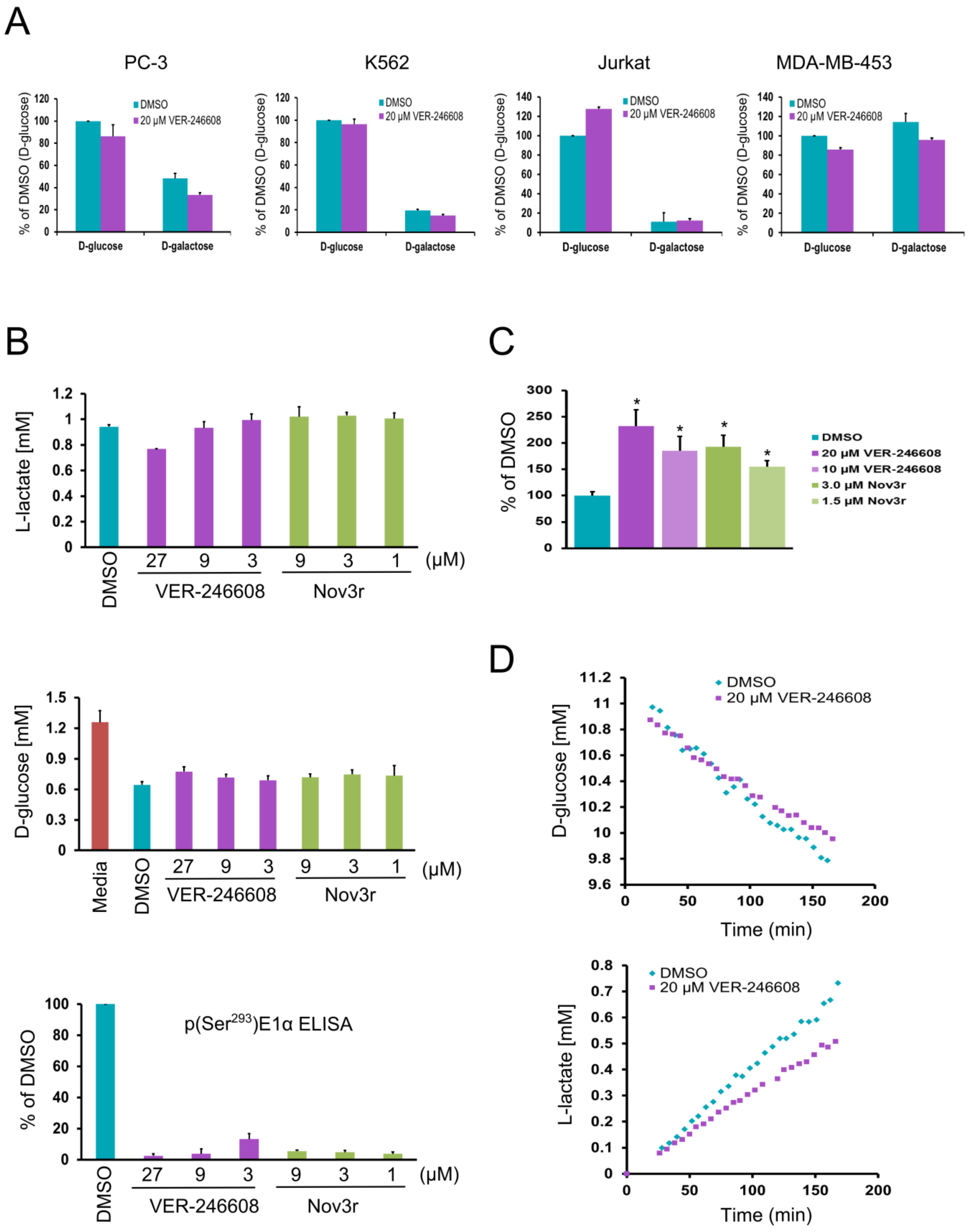
A
PC-3
K562
Jurkat
MDA-MB-453
% of DMSO (D-glucose)
DMSO
20 µM VER-246608
D-glucose
D-galactose
B
L-lactate [mM]
DMSO
VER-246608
Nov3r
(µM)
D-glucose [mM]
Media
% of DMSO
p(Ser293)E1α ELISA
C
% of DMSO
DMSO
20 µM VER-246608
10 µM VER-246608
3.0 µM Nov3r
1.5 µM Nov3r
D
D-glucose [mM]
Time (min)
L-lactate [mM]

Supplementary Figure S2: Effect of VER-246608 on Warburg metabolism in non-transformed and additional cancer cell lines. A, The indicated cell lines were seeded into 96 well plates and the following day treated with either DMSO or 20 µM VER-246608 in the relevant media containing either 2 g/L D-glucose or D-galactose for 96 h. Cell mass was determined using the sulforhodamine B assay. Results are expressed as the percentage change vs DMSO control values for cells incubated in D-glucose containing media. B, HFF cells were seeded into 96 well plates at a density of $1x10^{4}$ cells per well in 96 well plates. The following day the cells were treated with the indicated concentrations of VER-246608 and Nov3r (in DMEM media containing 0.25 g/L D-glucose and 2% dialyzed FCS) for 6 h, followed by the removal of cell culture media for analysis of L-lactate and D-glucose. At the end of the experiment the remaining media was aspirated and the cells transferred to a -80ºC freezer for subsequent analysis of p(Ser^{293})E1α levels by MSD ELISA. C. K562 cells were pre-incubated with DMSO, 20 µM VER-246608 or 1.5 µM Nov3r for 2 h followed by analysis of pyruvate dehydrogenase (PDH) activity in cell lysates using a commercially available ELISA-based assay kit (Abcam). Results are expressed as a percentage of the PDH reaction rate in DMSO treated cells. D, Jurkat cells (5 X 10^{6}) were pre-treated in RPMI-media (containing 10% FCS) with either DMSO or 20 µM VER-246608, followed by re-suspension in glucose-free RPMI-1640 media supplemented with 2 g/L 1,6-^{13}C-D-Glucose followed by kinetic acquisition of ^{13}C-NMR spectra at 10 min intervals. Representative curves from duplicate treatments are shown. Experiments were performed in triplicate (A & C) or quadruplicate (B). Error bars represent standard deviation of the mean (*, $P < 0.01$).

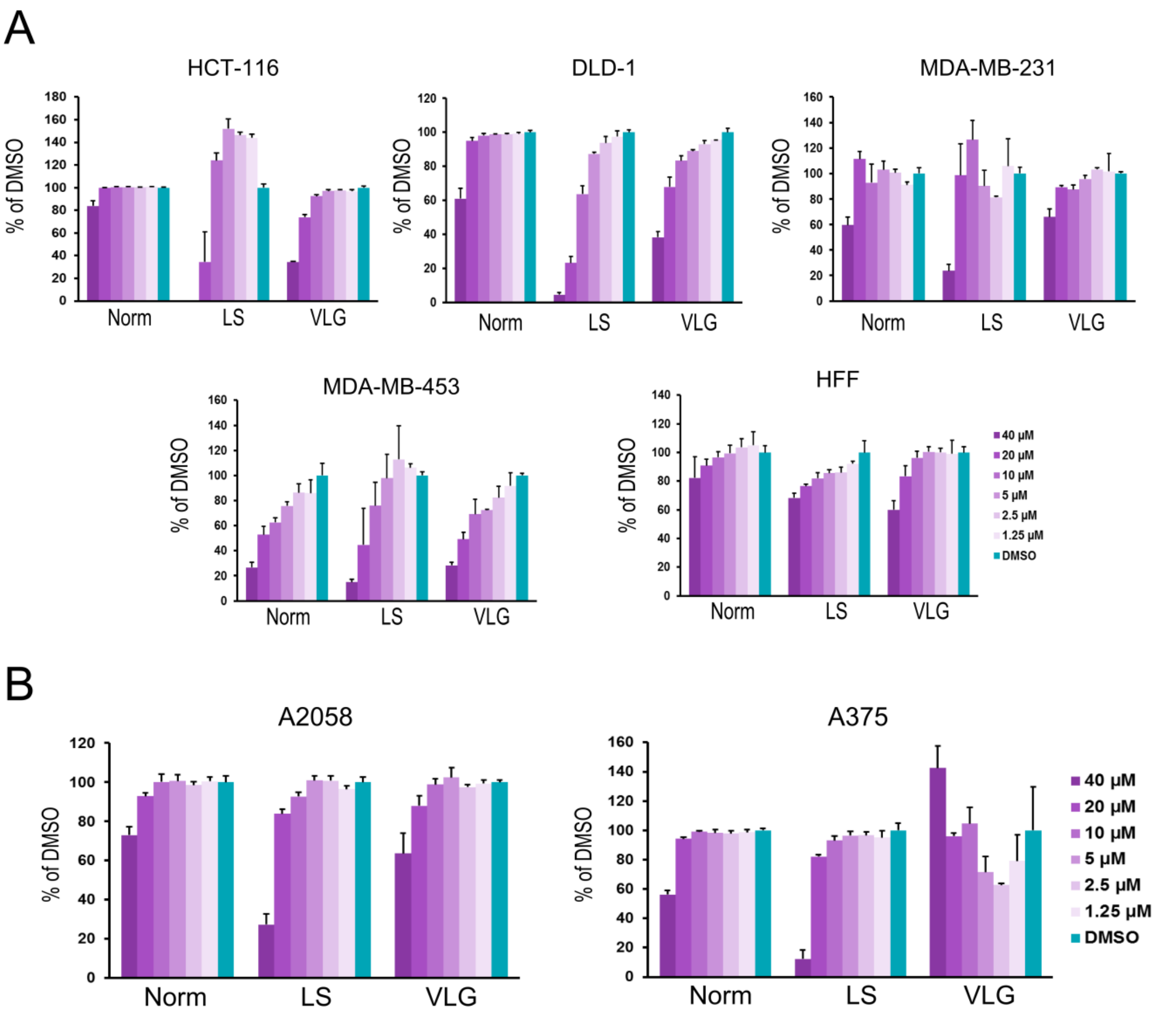

Supplementary Figure S3: Cytotoxicity of VER-246608 to cells lines bearing oncogenic KRAS(G13D) and BRAF(V600E) mutations. A and B, Cell lines harboring a KRAS(G13D) mutation (HCT-116, DLD-1 and MDA-MB-231), wild-type KRAS (MDA-MB-453) or a BRAF(V600E) mutation (A2058 and A375) were seeded into 96 well plates. The following day the cells were treated with the indicated concentrations of VER-246608 or DMSO in different culture conditions for 120 h. Cell mass was determined using the suforhodamine B assay. Norm = glucose-free media supplemented with 10% FCS and 2 g/L D-glucose; VLG (very low glucose) = 0.1 g/L D-glucose; LS (low serum) = 0.5% FCS. Experiments were performed in triplicate. Error bars represent standard deviation of the mean.

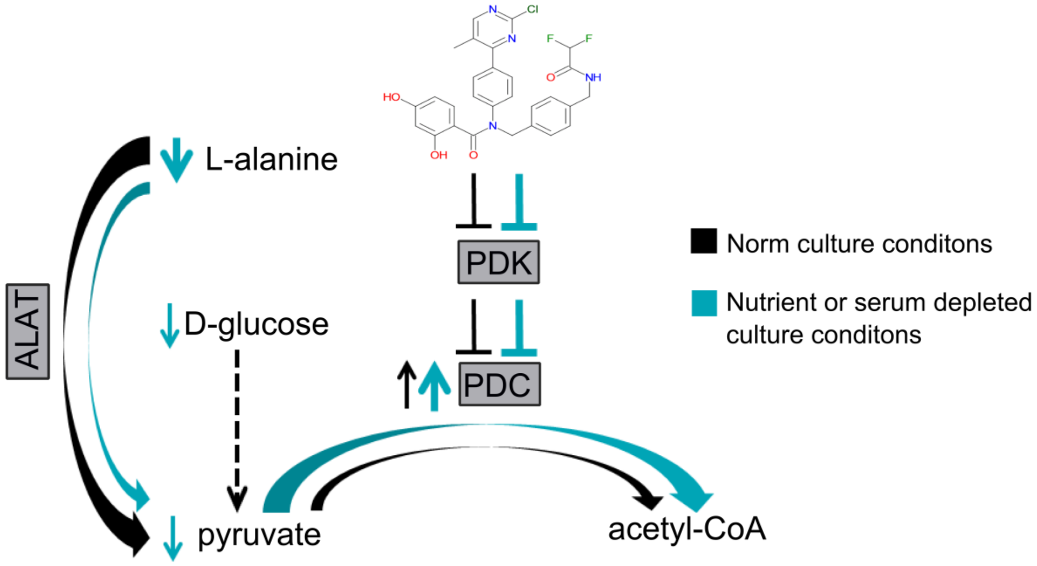

Supplementary figure S4: Schematic diagram illustrating a proposed mechanism for inhibition of cellular proliferation by VER-246608 under austere culture conditions. Under normal culture conditions L-alanine levels are sufficient to replenish pyruvate loss resulting from increased PDC activity through alanine aminotransferase (ALAT) catalysed deamination. However, under conditions of either nutrient or serum deprivation, increased cellular demand and/or reduced biosynthesis of L-alanine coupled with increased compound potency leads to insufficient L-alanine availability to maintain steady state pyruvate levels resulting in attenuation of glycolysis and cellular proliferation. Thickness of lines/arrows corresponds to the magnitude of the effect/change.

	GI$_{50}$(μM) ± SD							
	21% O_2				0.1% O_2			
Cell line	Norm	LG	VLG	LS	Norm	LG	VLG	LS
PC-3	26.2 ± 1.2	26.1 ± 2.6	14.8 ± 4.5	4.7 ± 1.6	36.0 ± 3.2	42.1 ± 4.9	32.3 ± 2.8	4.7 ± 1.6
SW-1088	> 80	> 80	40.5 ± 10.4	36.2 ± 8.2	> 80	> 80	> 80	> 80
UM-22B	> 80	45.7 ± 1.5	> 80	11.6 ± 2.8	> 80	45.1 ± 2.1	48.0 ± 5.8	14.6 ± 1.8
MEF	> 80	> 80	> 80	17.6 ± 2.0	60.8 ± 4.8	> 80	> 80	12.5 ± 2.3

Supplementary Table S1: Cytotoxicity of VER-246608 under different culture conditions. Norm = glucose-free media supplemented 10% FCS and 2 g/L D-glucose; LG (low glucose) = 0.5 g/L D-glucose; VLG (very low glucose) = 0.1 g/L D-glucose; LS (low serum) = 0.5% FCS. Values represent the average (± SD) of n=4 GI$_{50}$ determinations.

	GI_{50}(μM) ± SD							
	21% O_2				0.1% O_2			
Cell line	Norm	LG	VLG	LS	Norm	LG	VLG	LS
PC-3	25.7 ± 1.0	19.4 ± 1.3	8.1 ± 0.4	3.0 ± 0.8	33.9 ± 6.1	35.9 ± 2.1	25.5 ± 3.3	2.1 ± 0.5
SW-1088	> 80	46.2 ± 1.0	22.5 ± 1.7	23.5 ± 7.0	> 80	>80	51.6 ± 6.4	31.1 ± 3.0
UM-22B	48.0 ± 2.3	40.8 ± 0.6	20.8 ± 5.9	12.1 ± 1.2	31.7 ± 0.7	40.6 ± 1.8	40.5 ± 3.7	12.1 ± 0.7
MEF	> 80	50.0 ± 4.7	70.8 ± 1.3	11.4 ± 0.4	66.1 ± 4.3	> 80	64.5 ± 9.1	9.5 ± 2.0

Supplementary Table S2: **Cytotoxicity of VER-246520 under different culture conditions.** Norm = glucose-free media supplemented 10% FCS and 2 g/L D-glucose; LG (low glucose) = 0.5 g/L D-glucose; VLG (very low glucose) = 0.1 g/L D-glucose; LS (low serum) = 0.5% FCS. Values represent the average (± SD) of n=4 GI_{50} determinations.

Compound	VER-246608	VER-246520
Data collection statistics		
Resolution (Å)	2.60	2.50
Space group	P64	P64
Cell dimensions (Å)		
a =	108.9	108.8
b =	108.9	108.8
c =	84.4	84.4
No. molecules/asymmetric unit	1	1
Measured reflections	39992	37208
Unique reflections	17281	19916
Completeness: Overall / in hrb* (%)	98.3 / 97.7	99.1 / 99.1
Mean I/ I: Overall / in hrb	5.0 / 1.3	13.8 / 2.55
R_{merge}: Overall / in hrb (%)	0.125 / 0.531	0.10 / 0.58
Refinement statistics		
R_{free} (%)	0.252	0.249
R_{cryst} (%)	0.199	0.196
Rms Deviations:		
Bonds (Å)	0.014	0.022
Angles (°)	1.714	2.075
B Factor (Å^2)	11.828	7.937
PDB Code	4V25	4V26

Supplementary Table S3: Data collection and refinement statistics for PDK-2 in complex with compounds VER-246608 and VER-246520. R_{free} is the R factor calculated using 5% of the reflection data chosen randomly and omitted from the refinement process, whereas R_{cryst} is calculated with the remaining data used in the refinement. Rms bond lengths and angles are the deviations from ideal values; the rms deviation in B factors is calculated between covalently bonded atoms. *hrb: highest resolution bin.
